# Supplementary material for: A Chlamydia pneumoniae adhesin induces phosphatidylserine exposure on host cells
Source: Nat Commun. 2019 Oct 11;10:4644. doi: 10.1038/s41467-019-12419-8 (PMC6789132; doi:10.1038/s41467-019-12419-8)
Supplement: Supplementary file 1 — Supplementary Information [file 41467_2019_12419_MOESM1_ESM.pdf]

Supplementary Information

A *Chlamydia pneumoniae* adhesin induces phosphatidylserine exposure on host cells

Galle et al

Supplementary Content:

Supplementary Figures page 2

Primer List page 9

Supplementary references page 9

Supplementary Figures:

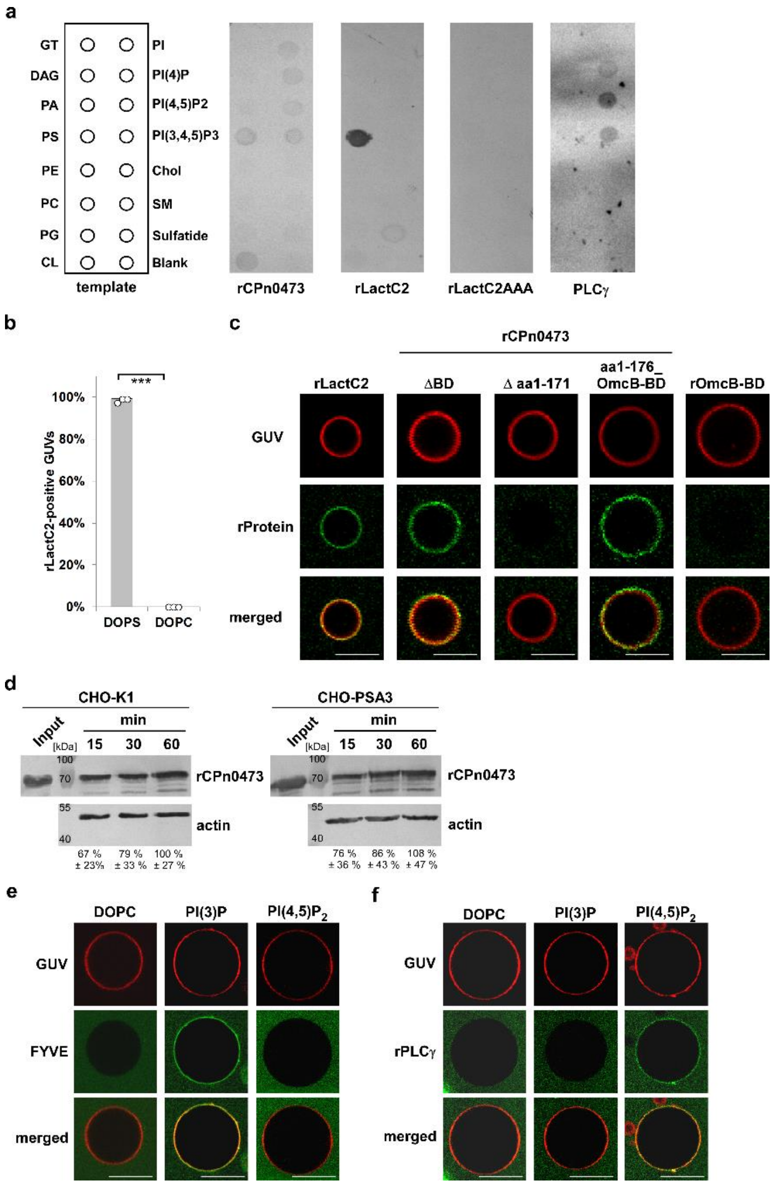

Supplementary Figure 1: CPn0473 binds to proteins on the host cell and to membrane phospholipids

a, Binding of rCPn0473, rLactadherin and rPLCγ (1 µg/ml) to membrane lipid strips. Binding affinity was analyzed using a monoclonal anti-His (rCPn0473) or anti-GST antibody (rPLCγ, rLactC2). The lipids which are spotted (100 pmol) can be identified in the template (left).

b, Binding efficiency of rLactC2 to giant unilamellar vesicles (GUVs). GUVs consisting of phosphatidylcholine (DOPC) or DOPC and phosphatidylserine (DOPS, 20 mol% were incubated with DyLight488-labeled rLactC2 (at least 60 GUVs counted. Mean ± s.d. (n=3). *** P<0.001, ** P<0.01 and * P<0.05, n.s. not significant (P>0.05) (Student's two-sample t-test).

c, Binding of rCPn0473 deletion variants to giant unilamellar vesicles (GUVs). Scale bars: 5 µm.

d, Binding of rCPn0473 (100 µg/ml) to wild-type and PS-deficient CHO cells quantified by western blotting (n=3).

e,f, Binding of rFYVE (**e**) and rPLCγ (**f**) to GUVs containing PI(3)P or PI(4,5)P2 (10 mol%). Scale bars: 10 µm. Source data are provided as a Source Data file

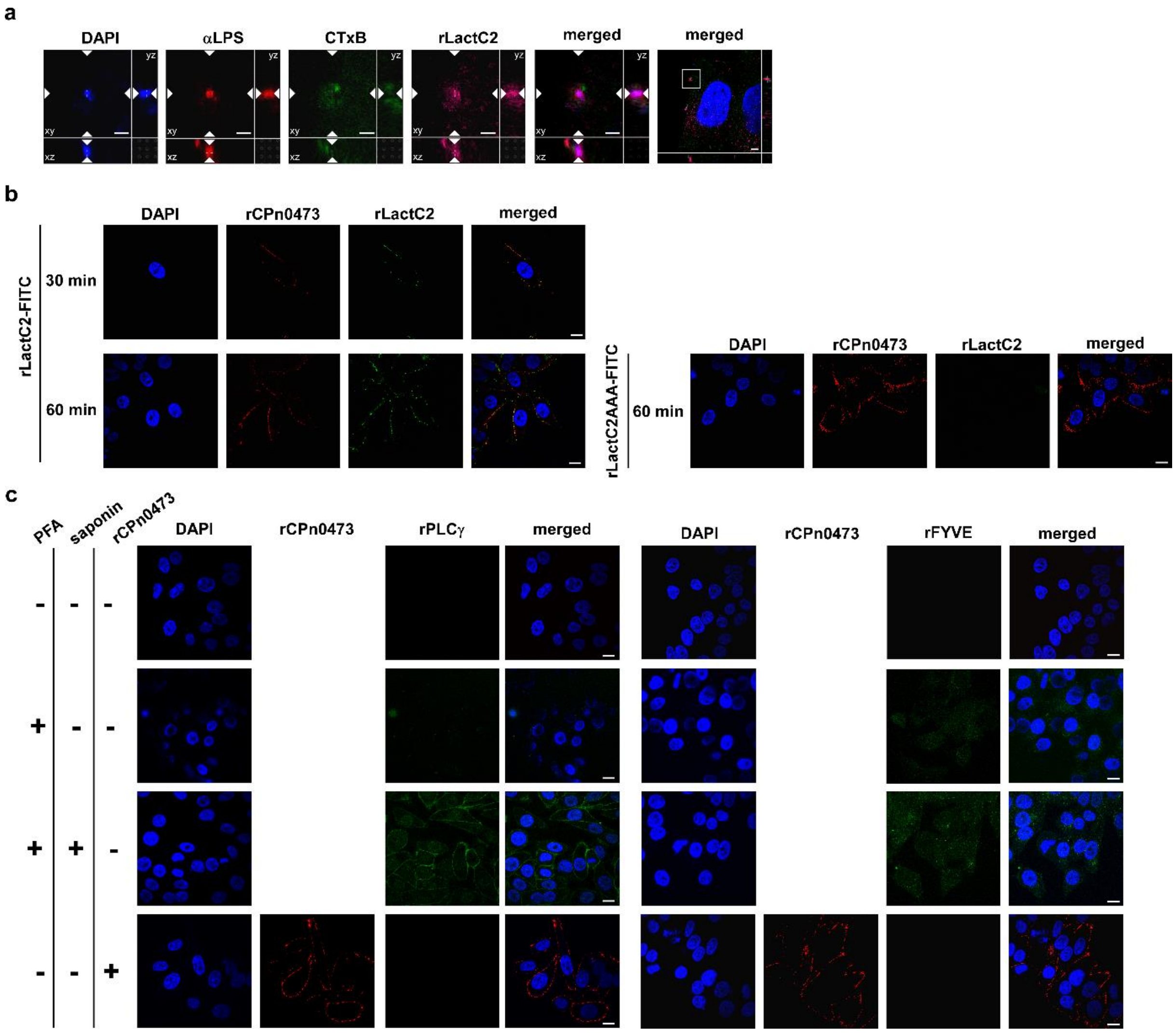

Supplementary Figure 2: Binding of *Cpn* or rCPn0473 to host cells induces phosphatidylserine (PS) externalization

a, Co-staining of PS, lipid rafts and *C. pneumoniae* early in infection (MOI=10). Liquid ordered phases (lo phases) are stained with the B subunit of cholera toxin (CTxB). Externalized PS was detected with rLactC2. The boxed area on the right is shown at higher magnification on the left. Scale bars: 2.5 µm.

b, PS externalization induced by rCPn0473 (100 µg/ml) and detected by rLactC2 or a rLactC2 mutant. Scale bars: 10 µm.

c, Co-staining of different phosphoinositides in the absence or presence of rCPn0473. Human HEp-2 cells were treated with or without rCPn0473 (100 µg/ml, 60 min at 37 °C) in DMEM medium. PI(4,5)P2 was detected by rPLCγ, and PI(3)P was detected by rFYVE prior to fixation with PFA. As a control, cells were fixed with PFA (10 min, 4 °C) and permeabilized with 2 % saponin (30 min, 4 °C) prior to

incubation with the respective lipid biosensor, to ensure that the latter had access to intracellular PIP. Scale bars: 10 µm.

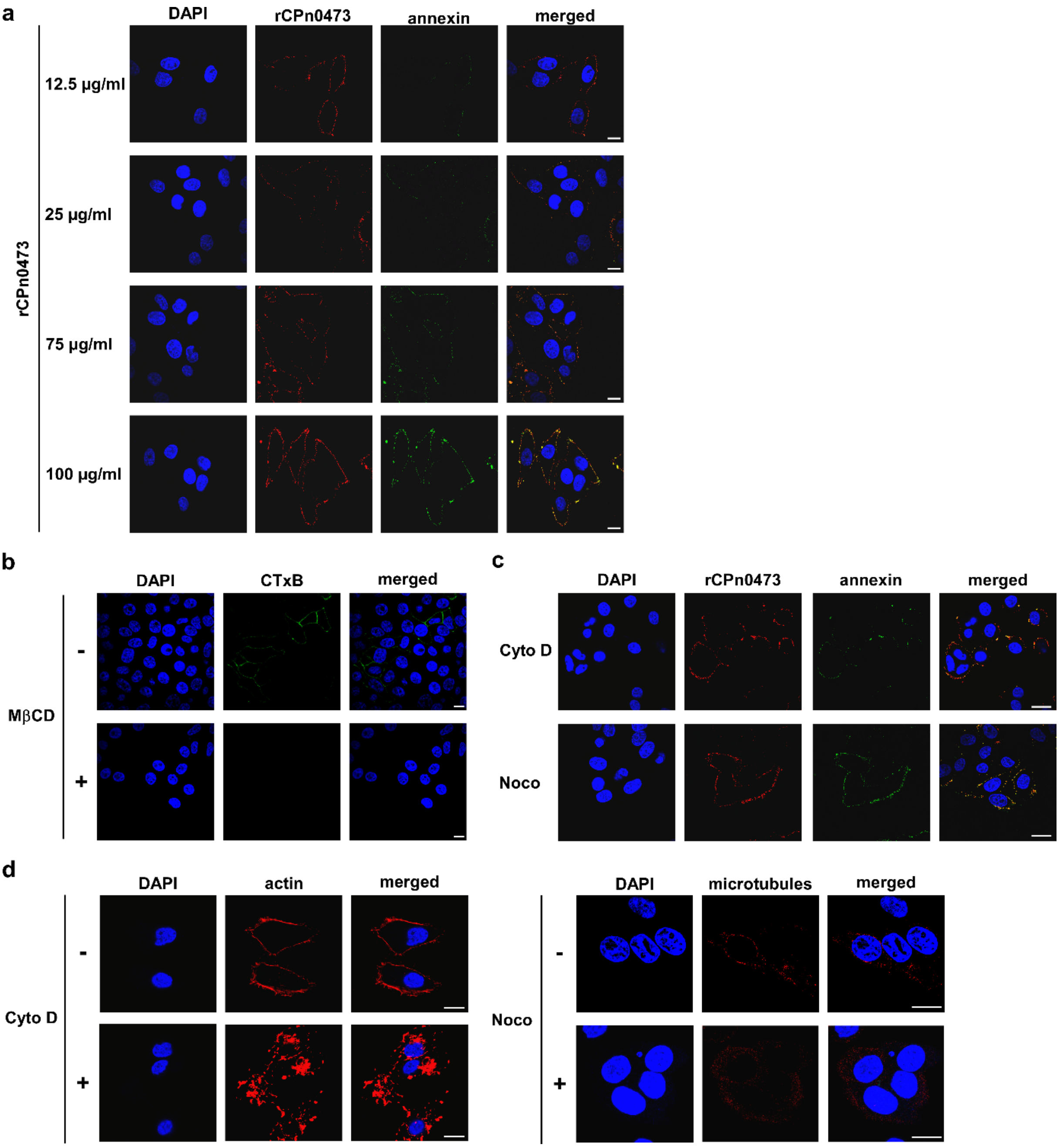

Supplementary Figure 3: CPn0473-induced PS externalization is not affected by the disruption of host cytoskeleton

a, rCPn0473-induced PS externalization detected by annexin prior to cell fixation. Scale bar: 10 µm

b, Staining for liquid-order phases (lo) in methyl-beta-cyclodextrin-treated (MβCD, 5 mM) cells by FITC-labelled Cholera toxin subunit B (CTxB). Scale bars: 10 µm.

c, PS externalization induced by rCPn0473 in cytoskeleton-depleted cells. HEp-2 cells were incubated with nocodazole (Noco, 10 µM) or with cytochalasin D (Cyto D, 20 µM). PS was stained using Annexin. Scale bars: 10 µm.

d, Staining for the host cytoskeleton in cytochalasin D and nocodazole-treated cells. Actin was stained with rhodamine phalloidin and microtubules were stained with tubulin-specific antibodies. Scale bars: 10 µm.

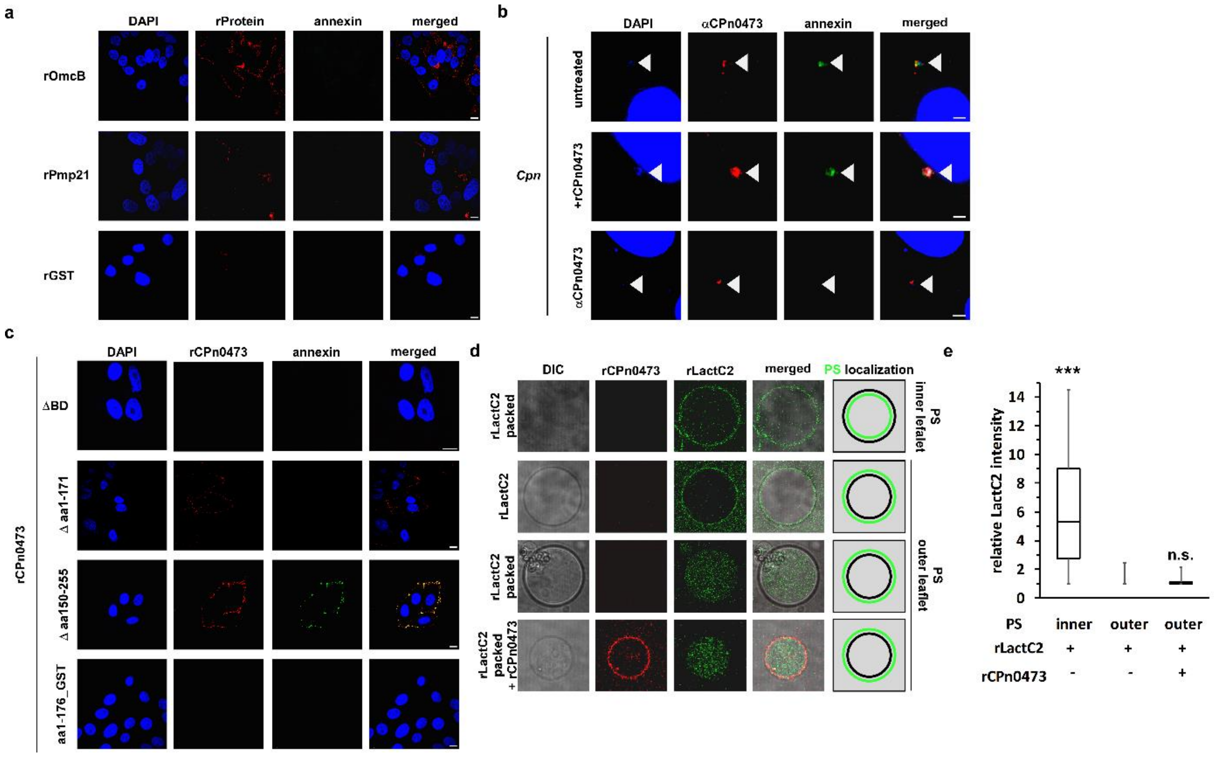

Supplementary Figure 4: Phosphatidylserine (PS) externalization by recombinant CPn0473

a-c, PS externalization assay with recombinant protein variants. HEp-2 cells were incubated with the indicated proteins (100 µg/ml) for 60 min at 37 °C prior to incubation with annexin to visualize externalized PS. **a,** PS externalization assay with recombinant adhesins (rOmcB and rPmp21) and a non-adhesin protein (rGST) on HEp-2 cells. HEp-2 cells were incubated with the indicated proteins (100 µg/ml) for 60 min at 37 °C prior to incubation with annexin to visualize externalized PS. Scale bars: 10 µm. **b,** Representative images of cells infected with *C. pneumoniae* EBs pre-treated with PBS (upper row) or CPn0473-specific antibodies (middle row) or coated with rCPn0473 (lower row). Scale bars: 2.5 µm. **c,** PS externalization assay with recombinant CPn0473 constructs on HEp-2 cells. HEp-2 cells were incubated with the indicated CPn0473 construct (100 µg/ml) for 60 min at 37 °C prior to incubation with annexin to visualize externalized PS. Scale bars: 10 µm.

d,e, Asymmetric GUVs were prepared based on [1]. In the presence or absence of rCPn0473, FITC-labeled, recombinant rLactC2, as a marker for PS, was packed into (packed) or incubated with GUVs bearing PS predominantly in the inner (PS_{inn}-GUV) or outer (PS_{out}-GUV) leaflet for 30 min at RT. Representative views are shown in **d**. Scale bar: 10 µm. rLactC2 binding was quantified using ImageJ software. Background intensity was set to 1 (at least 10 GUVs per experiment analyzed) (n=3) (**e**). Data

from GUVs, with luminal rLactC2, are represented as a box and whisker plot. The center line represents the mean score, the upper and lower quartiles contain the mean 50 % of the data, while the whiskers show the minima and maxima of all data (n=3).

*** P<0.001, ** P<0.01 and * P<0.05, n.s. not significant (P>0.05) (Student's two-sample t-test). Source data are provided as a Source Data file

Primer list

Supplementary Table 1: PCR primers

Internal number	Name	sequence (5`→3`)
**C-795**	Sequencing fwd	TTTCGTCTTCACCTCGAGAAA
**C-1292**	Sequencing rev	CAATAAAAAACGCCCGGCGG
**C-1313**	CPn0473 fwd	ATTTCACACAGAATTCATTAAAGAGGAGAAATTAACT ATGGCAGTTGGTGGCGTAGG
**C-1819**	CPn0473-IED_OmcB-BD rev	TAGGAACAGGTGCTGGCTTTGTTTCCGCACT GGATCCCAGAGGCTCTCCTCCTTCAGAAC
**C-2004**	CPn0473 ΔIED fwd	ATTTCACACAGAATTCATTAAAGAGGAGAAATTAACT ATG GAAGGAGGAGAGCCTTCTTC
**C-2006**	CPn0473-rev	CAGGAGTTCAATGGTGATGGTGATGATGGTGGTGATG GTG CTGTCCCTCTGGAGCAAATG
**C-2635**	CPn0473_Cys-N_fwd	ATTTCACACAGAATTCATTAAAGAGGAGAAATTAACT ATG TGT GCAGTTGGTGGCGTAGG
**C-2636**	CPn0473_Cys-C_rev	GAGTTCAATGGTGATGGTGATGGTGATGGTGATGGTG ACA CTGTCCCTCTGGAGCAAATG
**C-2766**	CPn0473-N_OmcB-BD rev	TAGGAACAGGTGCTGGCTTTGTTTCCGCACTGGATCC CAG CTGAACTGTAGACGTCGCTC
**C-2767**	OmcB-BD_CPn0473-N fwd	TTTTTTATCAGGAGTTCGAGGAGCGACGTCTACAGTT CAG CTGGGATCCAGTGCGGAAAC
**C-2768**	OmcB-BD_pFT25 rev	CAGGAGTTCAATGGTGATGGTGATGATGGTGGTGATG GTG TCTTCCGTAGCAAGACTCTT
**C-2295**	CPn0473_ΔBD-fwd	ATTTCACACAGAATTCATTAAAGAGGAGAAATTAACT ATG GAAGGAGGAGAGCCTTCTTC
**C-3098**	CPn0473 ΔTM rev	ATTGTGTATTACTTTCTGATGCAAGATCATCTACAGCT GA TCCCTCTACCCCACCTGTTT
**C-3099**	CPn0473 ΔTM fwd	AGAGACTGCGGAAGCTCCCGAAACAGGTGGGGTAGA GGGA TCAGCTGTAGATGATCTTGC

Supplementary references

1 Pautot, S., Frisken, B. J. & Weitz, D. A. Engineering asymmetric vesicles. *Proc Natl Acad Sci U S A* **100**, 10718-10721 (2003).
