# Supplementary material for: A Chlamydia pneumoniae adhesin induces phosphatidylserine exposure on host cells
Source: Nat Commun. 2019 Oct 11;10:4644. doi: 10.1038/s41467-019-12419-8 (PMC6789132; doi:10.1038/s41467-019-12419-8)
Supplement: Supplementary file 3 — Reporting Summary [file 41467_2019_12419_MOESM3_ESM.pdf]

nature research

Corresponding author(s): Johannes Hegemann

Last updated by author(s): Aug 23, 2019

Reporting Summary

Nature Research wishes to improve the reproducibility of the work that we publish. This form provides structure for consistency and transparency in reporting. For further information on Nature Research policies, see Authors & Referees and the Editorial Policy Checklist.

Statistics

For all statistical analyses, confirm that the following items are present in the figure legend, table legend, main text, or Methods section.

n/a	Confirmed	
☐	☒	The exact sample size (*n*) for each experimental group/condition, given as a discrete number and unit of measurement
☐	☒	A statement on whether measurements were taken from distinct samples or whether the same sample was measured repeatedly
☐	☒	The statistical test(s) used AND whether they are one- or two-sided<br>*Only common tests should be described solely by name; describe more complex techniques in the Methods section.*
☒	☐	A description of all covariates tested
☒	☐	A description of any assumptions or corrections, such as tests of normality and adjustment for multiple comparisons
☐	☒	A full description of the statistical parameters including central tendency (e.g. means) or other basic estimates (e.g. regression coefficient) AND variation (e.g. standard deviation) or associated estimates of uncertainty (e.g. confidence intervals)
☐	☒	For null hypothesis testing, the test statistic (e.g. F, t, r) with confidence intervals, effect sizes, degrees of freedom and P value noted<br>*Give P values as exact values whenever suitable.*
☒	☐	For Bayesian analysis, information on the choice of priors and Markov chain Monte Carlo settings
☒	☐	For hierarchical and complex designs, identification of the appropriate level for tests and full reporting of outcomes
☒	☐	Estimates of effect sizes (e.g. Cohen's d, Pearson's r), indicating how they were calculated

Our web collection on statistics for biologists contains articles on many of the points above.

Software and code

Policy information about availability of computer code

Data collection	NIS Element Software (Nikon) was used to collect microscopic data.<br>ABI PRISM 7000 Sequence Detektion System (Applied Biosystems) was used for qRT PCR analysis.
Data analysis	NIS Element Software and ImageJ software was used to quantify fluorescence signal of microscopic picture.<br>ImageJ software was used to quantify Western blots and fluorescence intensity.

For manuscripts utilizing custom algorithms or software that are central to the research but not yet described in published literature, software must be made available to editors/reviewers. We strongly encourage code deposition in a community repository (e.g. GitHub). See the Nature Research guidelines for submitting code & software for further information.

Data

Policy information about availability of data

All manuscripts must include a data availability statement. This statement should provide the following information, where applicable:

- Accession codes, unique identifiers, or web links for publicly available datasets
- A list of figures that have associated raw data
- A description of any restrictions on data availability

All data generated during this study are included in this publication and documented in the extended data set.

Field-specific reporting

Please select the one below that is the best fit for your research. If you are not sure, read the appropriate sections before making your selection.

☒ Life sciences ☐ Behavioural & social sciences ☐ Ecological, evolutionary & environmental sciences

For a reference copy of the document with all sections, see nature.com/documents/nr-reporting-summary-flat.pdf

Life sciences study design

All studies must disclose on these points even when the disclosure is negative.

Sample size	No sample size calculation was performed.
Data exclusions	No data were excluded from the analysis.
Replication	All attempts of replication were successful and are included into the manuscript.
Randomization	This is not relevant to this study.
Blinding	Blinding was either not relevant or not possible to the study. In order to assure the functionality of each assay, a positive and negative control in every sample size had to be used. Furthermore, to calibrate the analysis system (e.g. microscope settings), control samples were used prior to the test-sample.

Reporting for specific materials, systems and methods

We require information from authors about some types of materials, experimental systems and methods used in many studies. Here, indicate whether each material, system or method listed is relevant to your study. If you are not sure if a list item applies to your research, read the appropriate section before selecting a response.

Materials & experimental systems

n/a	Involved in the study	
☐	☒	Antibodies
☐	☒	Eukaryotic cell lines
☒	☐	Palaeontology
☒	☐	Animals and other organisms
☒	☐	Human research participants
☒	☐	Clinical data

Methods

n/a	Involved in the study	
☒	☐	ChIP-seq
☒	☐	Flow cytometry
☒	☐	MRI-based neuroimaging

Antibodies

Antibodies used

Anti-PentaHIS from Quiagen (#34660)
Anti-GST from Santa Cruz (Z-5) (#2309)
Anti-CPn0473 were produced in rabbit by Eurogentec (Animal ID 6712)
Anti-Pmp21 were produced in rabbit by Eurogentec (Animal ID 2615)
Anti-Tubulin from Acris (Clone TU-01) (#BM753S)
Anti-ß- actin from Sigma Aldrich (Clone AC-15) (#A1978)
Anti-EGFR from Invitrogen (# MA5-13269)
Anti-MOMP generated by G. Zhong (University of Texas Health Science Center at San Antonio)
Anti-LPS (Pathfinder) from BioRad
Anti-Chlamydia LPS from US Biomol (#C4250-51F) (clone 5F153)
Anti-Caspase-3 (Institute for Molecular Medicine, HHU Düsseldorf)
Anti-PARP (Institute for Molecular Medicine, HHU Düsseldorf)
AP-coupled anti-Mouse from Promega (#S372)
AP-coupled anti-Rabbit from Promega (#S3731)
AP-coupled anti-Goat from Santa Cruz (#sc-2355)
Alexa488-conjugated anti-rabbit from Life Technologies (#A1108)
Alexa594-conjugated anti-rabbit from Life Technologies (#A11012)
Cy5 conjugated anti-rabbit from Life Technologies (#A10523)
Alexa488-conjugated anti-mouse antibodies from Life Technologies (#A1101)
Alexa594-conjugated anti-mouse antibodies from Life Technologies (#A21203)

Validation

Antibodies produce by Eurogentec were validated elsewhere:
Fechtner et al (2016) Cellular microbiology
Moelleken et al (2010) Mol Microbiol IP Mass Spectrometry

Commercially available antibodies were validated for on-target binding during post immunization screening solely by IP Mass Spectrometry, Western blot (WB) or immunohistochemistry (IHC).

Eukaryotic cell lines

Policy information about cell lines

Cell line source(s)	HEp-2 (Human epithlial Larynxcarzinom-cell line, (ATCC Nr.: CCL-23)).<br>CHO-K1 (Wildtype Chinese hamster ovary cells) from Osamu Kuge (Kyushu University, Japan).<br>CHO-PSA3 (PS-deficient Chinese hamster ovary cells) from Osamu Kuge (Kyushu University, Japan).
Authentication	None of the cell lines used were autheniticated.
Mycoplasma contamination	HEp-2 cells were tested negative for mycoplasma contamination.<br>CHO cells were not tested for mycoplasma contamination.
Commonly misidentified lines<br>(See ICLAC register)	*Name any commonly misidentified cell lines used in the study and provide a rationale for their use.*
