# Supplementary material for: A Chlamydia pneumoniae adhesin induces phosphatidylserine exposure on host cells
Source: Nat Commun. 2019 Oct 11;10:4644. doi: 10.1038/s41467-019-12419-8 (PMC6789132; doi:10.1038/s41467-019-12419-8)
Supplement: Supplementary file 4 — Source Data [file 41467_2019_12419_MOESM4_ESM.pdf]

Source Data

A *Chlamydia pneumoniae* adhesin induces phosphatidylserine exposure on host cells (Galle et al)

The file contains raw data and uncropped blots for the following figures:

- **Figure 1a, b, d, e**
- **Figure 2a, b, d**
- **Figure 3b, c, e**
- **Figure 4a,c, d, g**
- **Supp. Figure 1a, b, d**
- **Supp. Figure 4e**

<u>Figure 1</u>

a)

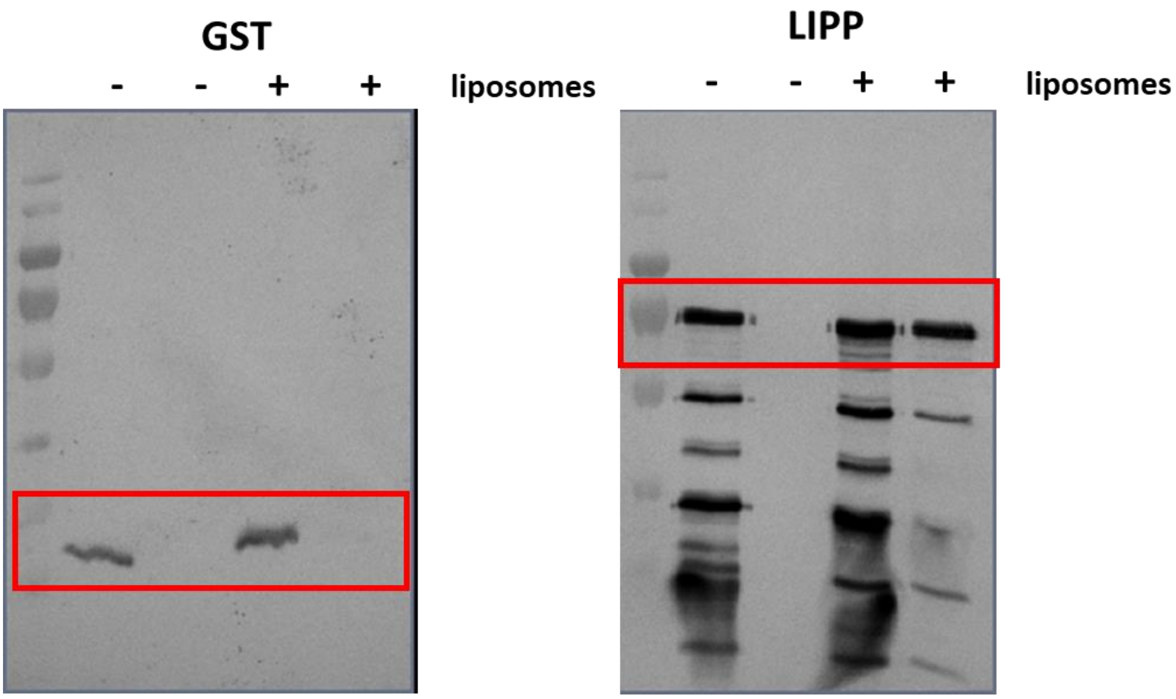

Antibodies used:

Anti-HIS

b)

		**rLIPP-488**			
		(+)	(-)	total	% LIPP positive
**Assay 1**	DOPC	0	79	79	0
	+Chol	1	79	80	0,0125
	DOPS	33	42	75	0,44
	+Chol	41	40	81	0,50617284
	DOPA	14	88	102	0,137254902
	+Chol	14	90	104	0,134615385
	PI(3,4)P_2	0	97	97	0
	+Chol	10	66	76	0,131578947
		rLIPP-488			
		(+)	(-)	total	% LIPP positive
**Assay 2**	DOPC	1	80	81	0,012345679
	+Chol	4	71	75	0,053333333
	DOPS	33	51	84	0,392857143
	+Chol	50	49	99	0,505050505
	DOPA	23	77	100	0,23
	+Chol	25	80	105	0,238095238
	PI(3,4)P_2	0	101	101	0
	+Chol	8	76	84	0,095238095
		rLIPP-488			
		(+)	(-)	total	% LIPP positive
**Assay 3**	DOPC	0	125	125	0
	+Chol	2	76	78	0,025641026
	DOPS	32	48	80	0,4
	+Chol	36	40	76	0,473684211
	DOPA	12	89	101	0,118811881
	+Chol	14	83	97	0,144329897
	PI(3,4)P_2	1	85	86	0,011627907
	+Chol	9	73	82	0,109756098

d)

**rLIPP FL 20mol% DOPS**				**rLIPP FL 5mol% DOPS**			
**picture ID**	**+**	**-**	**total GUVs**	**picture ID**	**+**	**-**	**total GUVs**
**1**	4	2	6	1	1	0	1
**2**	7	5	12	2	3	4	7
**3**	2	14	16	3	1	0	1
**4**	2	0	2	4	2	3	5
**5**	12	4	16	5	1	2	3
**6**	10	0	10	6	3	1	4
**7**	10	2	12	7	5	5	10
**8**	11	2	13	8	3	2	5

**9**	14	2	16	9	2	2	4
**10**	21	1	22	10	2	0	2
**11**	1	3	4	11	1	0	1
**12**	2	6	8	12	2	3	5
**13**	3	11	14	13	5	6	11
**14**	4	3	7	14	7	7	14
**15**	5	12	17	15	1	0	1
**16**	6	11	17				
**17**	7	10	17				
**18**	8	1	9				
**19**	9	12	21				
**20**	10	9	19				
**21**	17	3	20				
**22**	17	0	17				
**23**	6	5	11				
**24**	9	6	15				
**25**	9	2	11				
**26**	11	0	11				
**27**	9	1	10				
**28**	14	0	14				

e)

raw data for rLIPP FL from d) (rLIPP FL 20mol% DOPS)

rLIPPΔBD				rLIPPΔIED			
**picture ID**	**+**	**-**	**total GUVs**	**picture ID**	**+**	**-**	**total GUVs**
**1**	1	0	1	1	0	12	12
**2**	1	0	1	2	0	9	9
**3**	2	1	3	3	0	12	12
**4**	5	0	5	4	0	11	11
**5**	7	0	7	5	0	15	15
**6**	1	0	1	6	0	16	16
**7**	3	0	3	7	0	10	10
**8**	12	2	14	8	0	17	17
**9**	11	1	12	9	1	11	12
**10**	9	0	9	10	0	5	5
**11**	8	1	9	11	0	14	14
				12	0	5	5
				13	0	1	1
				14	0	12	12

rOmcB-BD_GST				rLIPP-IED_OmcB-BD			
**picture ID**	**+**	**-**	**total GUVs**	**picture ID**	**+**	**-**	**total GUVs**
**1**	0	13	13	1	12	2	14
**2**	0	14	14	2	7	0	7
**3**	0	15	15	3	18	1	19
**4**	0	9	9	4	16	1	17
**5**	0	18	18	5	58	1	59
**1**	0	5	5	6	64	6	70
**2**	1	6	7	7	50	4	54
**3**	1	10	11	8	36	7	43
**4**	0	2	2				
**5**	1	6	7				
**6**	1	10	11				
**7**	0	9	9				
**8**	0	14	14				

Figure 2

a)

Assay 1

	CHO-K1			CHO-PSA		
**#**	**1**	**2**	**3**	**1**	**2**	**3**
**visual fields**						
**1**	4	3	3	1	1	3
**2**	4	1	3	1	2	3
**3**	6	4	4	2	3	3
**4**	4	2	5	2	4	2
**5**	5	4	5	3	1	3
**6**	4	3	5	1	2	1
**7**	5	3	3	2	1	2
**8**	4	2	3	1	3	1
**9**	3	3	3	3	2	3
**10**	4	2	4	4	2	1
**Ø**	4	3	4	2	2	2
**Ø**	4					
**standardized**	1,19	0,75	1,06	0,56	0,58	0,61
**Ø**	1,00			0,58		

Assay 2

	CHO-K1			CHO-PSA		
**#**	**1**	**2**	**3**	**1**	**2**	**3**
**visual fields**						
**1**	3	4	3	2	1	1
**2**	3	5	3	3	1	1
**3**	4	4	6	2	2	3
**4**	5	5	4	2	2	3
**5**	7	4	5	1	2	2
**6**	3	4	5	3	1	3
**7**	4	5	4	2	2	2
**8**	6	4	4	3	2	1
**9**	5	6	6	1	1	2
**10**	7	3	7	2	3	3
**Ø**	5	4	5	2	2	2
**Ø**	5					
**standardized**	1,02	0,96	1,02	0,46	0,37	0,46
**Ø**	1,00			0,43		

Assay 3

	CHO-K1			CHO-PSA		
**#**	**1**	**2**	**3**	**1**	**2**	**3**
**visual fields**						
**1**	5	7	6	3	1	4
**2**	6	5	4	5	4	3
**3**	4	4	6	4	5	3
**4**	4	7	9	3	3	4
**5**	8	7	7	3	6	3
**6**	7	8	5	2	4	1
**7**	7	6	7	5	3	4
**8**	5	5	6	4	7	3
**9**	7	6	6	4	3	5
**10**	8	6	6	5	4	2
**Ø**	6	6	6	4	4	3
**Ø**	6					
**standardized**	0,99	0,99	1,01	0,62	0,65	0,52
**Ø**	1,00			0,60		

b)

The number of 16S gene copies relative to GAPDH gene copies was expressed as $2^{-\Delta\Delta CT}$. $2^{-\Delta\Delta CT}$ was used for the graph.

**Assay 1**						
		**delta CT2**	**delta delta CT**	**2^(-delta delta CT)**	**delta delta CT**	**2^(-delta delta CT)**
		**Ct GAPDH - Ct 16S**	**delta CT1 - delta CT2**		**delta CT (BSA) - delta CT (LIPP)**	
**CHO K1**	BSA	0,395	0	1,00	0	1,00
	LIPP	5,04	-4,645	25,02	-4,645	25,02
**CHO PSA 3**	BSA	-1,12	1,515	0,35	0	1,00
	LIPP	-1,101	1,496	0,35	-0,018	1,01
**Assay 2**						
		**delta CT2**	**delta delta CT**	**2^(-delta delta CT)**	**delta delta CT**	**2^(-delta delta CT)**
**CHO K1**	BSA	-3,405	0	1,00	0	1,00
	LIPP	1,544	-4,950	30,92	-4,950	30,92
**CHO PSA 3**	BSA	-1,305	-2,1005	4,29	0	1,00
	LIPP	-0,389	-3,0164	8,09	-0,915	1,89
**Assay 3**						
		**delta CT2**	**delta delta CT**	**2^(-delta delta CT)**	**delta delta CT**	**2^(-delta delta CT)**

**K1 -PS**	BSA	-3,746	0	1,00	0	1,00
	LIPP	1,006	-4,753	26,97	-4,753	26,97
**PSA 3 -PS**	BSA	-2,75	-0,996	2,00	0	1,00
	LIPP	-5,948	2,201	0,22	3,198	0,11

d)

Assay 1

C. pn.	1			2			3		
	total	PS-positive	% positive	total	PS-positive	% positive	total	PS-positive	% positive
1	9	7		8	7		8	5	
2	14	10		12	8		12	8	
3	16	13		11	9		13	12	
Σ	39	30	0,77	31	24	0,77	33	25	0,76

C. tr. E	1			2			3		
	total	PS-positive	% positive	total	PS-positive	% positive	total	PS-positive	% positive
1	19	4		18	2		14	3	
2	21	2		19	4		24	1	
3	22	2		23	4		21	1	
Σ	62	8	0,13	60	10	0,17	59	5	0,08

C. tr. LGV	1			2			3		
	total	PS-positive	% positive	total	PS-positive	% positive	total	PS-positive	% positive
1	22	3		22	2		19	1	
2	21	2		20	0		21	2	
3	19	1		18	2		19	2	
Σ	62	6	0,10	60	4	0,07	59	5	0,08

Assay 2

C. pn.	1			2			3		
	total	PS-positive	% positive	total	PS-positive	% positive	total	PS-positive	% positive
1	18	16		25	21		26	24	
2	27	23		17	15		21	21	
Σ	45	39	0,87	42	36	0,86	47	45	0,96

C. tr. E	1			2			3		
	total	PS-positive	% positive	total	PS-positive	% positive	total	PS-positive	% positive
1	25	4		18	2		18	5	
2	26	6		16	1		15	3	
Σ	51	10	0,20	34	3	0,09	33	8	0,24

	1			2			3		
C. tr. LGV	total	PS-positive	% positive	total	PS-positive	% positive	total	PS-positive	% positive
1	31	3		53	4		46	5	
2	35	2		18	0		34	6	
Σ	66	5	0,08	71	4	0,06	80	11	0,14

Assay 3

C. pn.	1			2			3		
	total	PS-positive	% positive	total	PS-positive	% positive	total	PS-positive	% positive
1	35	27		22	15		27	18	
2	48	35		42	31		29	22	
Σ	83	62	0,75	64	46	0,72	56	40	0,71

C. tr. E	1			2			3		
	total	PS-positive	% positive	total	PS-positive	% positive	total	PS-positive	% positive
1	26	4		57	6		50	4	
2	44	3		13	0		40	2	
Σ	70	7	0,10	70	6	0,09	90	6	0,07

	1			2			3		
C. tr. LGV	total	PS-positive	% positive	total	PS-positive	% positive	total	PS-positive	% positive
1	46	3		37	1		43	0	
2	50	4		43	1		35	2	
Σ	96	7	0,07	80	2	0,03	78	2	0,03

Figure 3

b)

		Assay		
	time point	1	2	3
**0 mM**	15	37,90136	90,207978	49,88871083
	30	236,9798	215,11936	309,1304348
	60	482,3881	300,00114	368,0335648
**1 mM**	15	87,80742	53,21259	81,49910434
	30	406,4836	222,26218	280,4296083
	60	132,6008	359,34395	144,2665256
**3 mM**	15	70,44883	62,381811	42,4609375
	30	489,8181	73,511352	252,6946429
	60	285,8388	65,527235	187,1859852

c)

Assay 1

12.5 ng/µl	#		
	1	2	3
(ROI)	(PS)	(PS)	(PS)
1	35333	25689	36664
2	28207	19104	37436
3	27330	19549	47980

25 ng/µl	#		
	1	2	3
(ROI)	(PS)	(PS)	(PS)
1	100874	98132	84802
2	107233	146219	68802
3	99967	146187	84114

50 ng/µl	#		
	1	2	3
(ROI)	(PS)	(PS)	(PS)
1	128082	332329	229609
2	86396	233369	166608
3	129842	346774	210609

100 ng/µl	#		
	1	2	3
(ROI)	(PS)	(PS)	(PS)
1	674755	675443	203303
2	514913	725910	116211
3	508754	595666	122825

Assay 2

12.5 ng/µl	#				
	1	2	3	4	5
(ROI)	(PS)	(PS)	(PS)	(PS)	(PS)
1	37803	36553	21449	49359	53634
2	34131	28224	47638	33638	55600
3	9811	66326	19176	56006	61944

25 ng/µl	#				
	1	2	3	4	5
(ROI)	(PS)	(PS)	(PS)	(PS)	(PS)
1	77246	46582	25325	132440	72955
2	100453	30308	13843	94897	46984
3	83778	69151	22253	106060	104651

50 ng/µl	#				
	1	2	3	4	5
(ROI)	(PS)	(PS)	(PS)	(PS)	(PS)
1	53910	112607	66026	115044	114825
2	56110	147274	76714	100356	68099
3	74752	126112	64909	77586	67224

100 ng/µl	#				
	1	2	3	4	5
(ROI)	(PS)	(PS)	(PS)	(PS)	(PS)
1	226626	172251	69732	121824	83150
2	105326	126238	76326	139707	92857
3	133360	239284	94314	132969	170149

Assay 3

12.5 ng/µl	#		
	1	2	3

(ROI)	(PS)	(PS)	(PS)
1	91312	137699	109984
2	149477	224128	195827
3	240059	215723	153192

25 ng/µl	#		
	1	2	3
(ROI)	(PS)	(PS)	(PS)
1	242854	124341	74866
2	212523	228589	79944
3	187333	287619	64899

50 ng/µl	#		
	1	2	3
(ROI)	(PS)	(PS)	(PS)
1	173432	237426	237759
2	82885	263395	161109
3	159164	179563	187823

100 ng/µl	#		
	1	2	3
(ROI)	(PS)	(PS)	(PS)
1	371138	368723	436972
2	431801	502839	397433
3	900795	519622	202097

e)

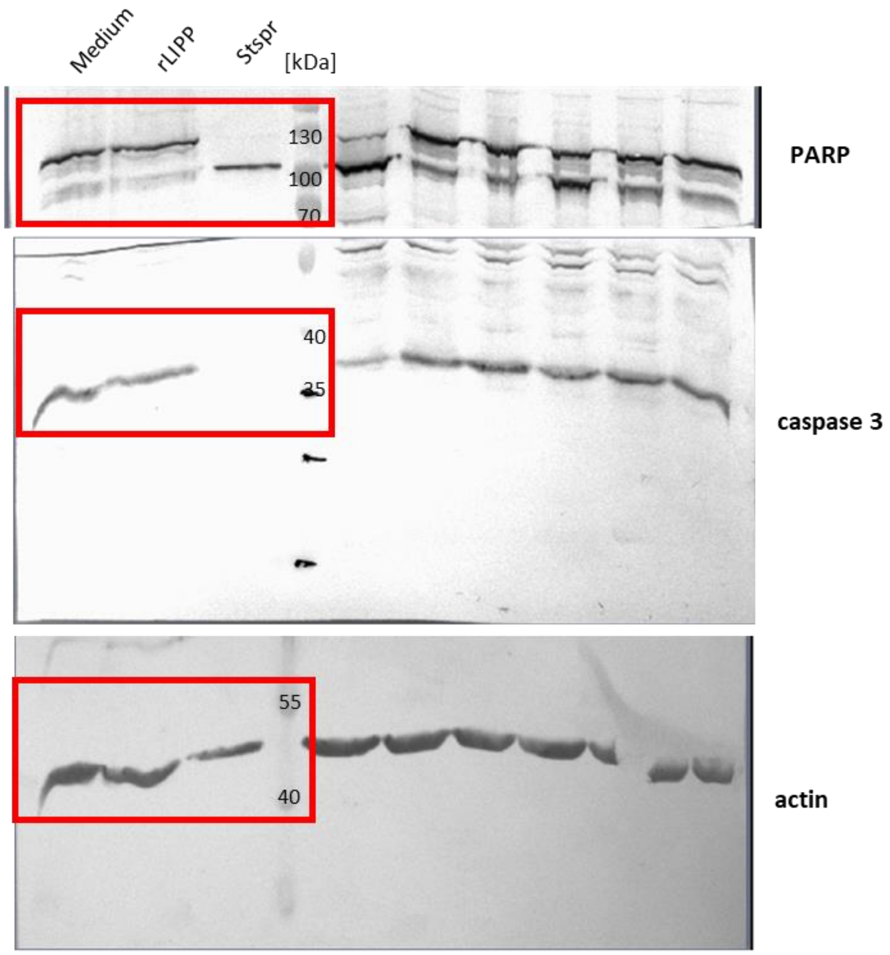
Medium
rLIPP
Stspr
[kDa]
130
100
70
PARP
40
35
caspase 3
55
40
actin

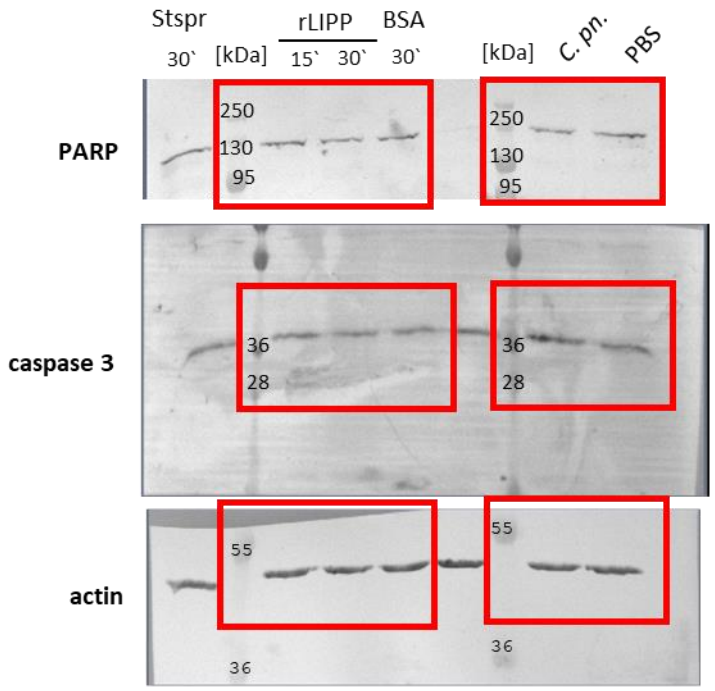
Stspr
30`
[kDa]
rLIPP
15`
30`
BSA
30`
[kDa]
C. pn.
PBS
PARP
250
130
95
250
130
95
caspase 3
36
28
36
28
actin
55
36
55
36

Antibodies used:

Anti PARP

Anti-caspase 3

Anti-Actin

f)

exemplary data set, displayed in the manuscript:

C.pn. + rLIPP														
#	1	2	3	4	5	6	7	8	9	10	11	12	13	
PS	761,85	918,63	144,23	154,16	760,44	1256,91	63,36	83,6	334,78	257,13	871,45	830,27	210,62	
#	14	15	16	17	18	19	20	21	22	23	24	25	26	27
PS	433,45	229,53	806,2	175,52	793,43	660,46	84,27	321,23	39,31	54,91	100,13	88,63	339,51	106,88

C.pn.												
#	1	2	3	4	5	6	7	8	9	10	11	12
PS	123,93	458,48	541,09	298,85	98,14	67,38	72,2	68,05	66,58	147,76	223,76	27,13
#	13	14	15	16	17	18	19	20	21	22	23	
PS	80,18	173,76	57,18	46,43	63,1	142,87	55,13	38,86	29,45	33,97	29,62	

g)

Assay 1

		#	untreated	anti-LIPP	anti-Pmp21-N
adherd C. pn.	DAPI (+) LPS (+) PS (-)	1	12	33	8
		2	18	13	15
		3	7	14	7
	DAPI (+) LPS (+) PS (+)	1	30	11	22
		2	47	14	25
		3	22	11	14

Assay 2

			untreated	anti-LIPP	anti-Pmp21-N
adherd *C. pn.*	DAPI (+) LPS (+) PS (-)	1	0	10	1
		2	3	4	2
	DAPI (+) LPS (+) PS (-)	1	29	67	59
		2	41	37	25

Figure 4

a)

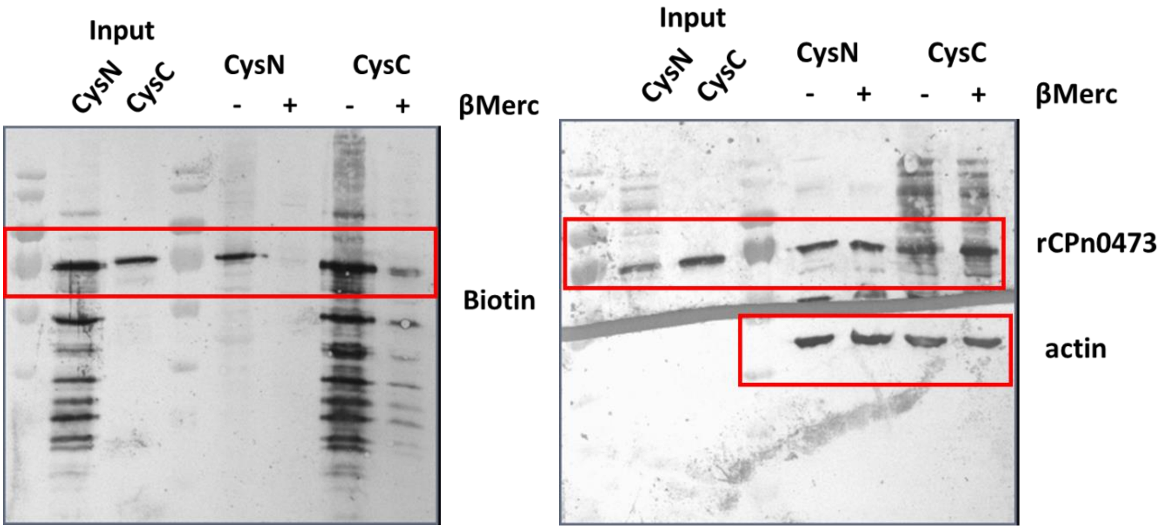

Antibodies used:

Anti-HIS (rCPn0473)

Anti-Actin (Actin)

Alkaline phosphatase-coupled- streptavidin (biotin)

c)

Full-length CF intensity

		Assay 1									Assay 2							
	Background	1	2	3	4	5	6	7	8	9	10	11	12	13	14	15	16	17
0	1			1,39837029	1,37511791	1,40584833	1,18930345	1,41264448	1,23546726		8,45195435	9,14458045	6,7533558	7,81160192	8,161965585	6,6410029		
1	1	1,408250155	1,40825016	1,40085769	1,36741008	1,39357089	1,2002198	1,38255707	1,23921016		8,48478591	9,40827982	8,12945694	1,70649543	7,053144461	7,05314446	4,32364235	
2	1	1,43357815	1,43357815	1,40005249	1,3453891	1,38123149	1,18704775	1,35723659	1,24998438		5,63164582	9,21360262	8,29452284	1,64796362	7,036915669	3,94870595	3,94870595	
3	1	1,441764227	1,44176423	1,38528239	1,32424935	1,36169499	1,17477645	1,3153202	1,24063536	1,27214545	5,55838883	2,59553403	8,5456747	1,5219203	7,298621746	4,16674383	4,16674383	
4	1	1,450719105	1,45071911	1,35178105	1,07967938	1,31713555	1,14804757	1,27500198	1,07836106	1,25040209	5,06795834	1,38589846	8,39522179	1,35435939	7,153106563	3,96358074	3,96358074	
5	1	1,435979317	1,43597932	1,1897873	0,99894813	1,30560223	0,94684644	1,14274005	1,07427291	1,26440173	3,04676899	1,17499365	6,1757556	1,11171583	7,038931824	1,84231341	1,84231341	
6	1	1,423775203	1,4237752	1,15015074	0,9717787	1,25937919	0,94350156	0,95528138	1,05546561	1,24249107	1,88041706	1,06904265	6,2374218	1,13819905	7,032492891	1,27719431	1,27719431	
7	1	1,410384369	1,41038437	1,14400745	0,95353535	1,12942188	0,93493803	0,95175872	1,0593309	1,2281109	1,43160305	1,05938931	5,49549618	1,05847328	6,961679389	1,25412214	1,25412214	7,74
8	1	1,400571132	1,40057113	1,1135572	0,93799691	0,96891763	0,93467558	0,95824822	1,04774781	1,21848777	1,1477246	1,00046996	0,92793922	0,995927	6,729928722	1,15242422	6,63875617	6,63875617
9	1	1,394186571	1,39418657	1,10011871	0,92752926	0,97978994	0,93714595	0,96704783	1,04256886	1,19874983	1,22257624	1,02097902	0,85438822	1,03537882	7,102412381	1,23664501	6,85256755	6,85256755
10	1	1,375006403	1,3750064	1,04645373	0,90946077	0,9812667	0,93308234	0,95521618	1,0219213	1,1850255	1,08226077	1,01335039	0,78175713	0,96842274	6,594531937	1,15844592	6,52610121	6,52610121
11	1	1,358378974	1,35837897	0,93726662	0,89419965	0,97208738	0,92982947	0,9579288	1,01328728	1,17469505	1,05269058	0,96172325	0,77338245	1,00992953	6,340086483	0,95091288	5,78155029	5,78155029
12	1	1,335586552	1,33558655	0,92112747	0,88478797	0,96811603	0,92542501	0,95715098	1,00243317	1,15840865	0,97739013	1,01227877	0,83673469	1,02938437	6,643830976	0,98856804	5,28715387	5,28715387
13	1	1,320823749	1,32082375	0,93208725	0,86768112	0,97249389	0,92262901	0,95222623	0,99153906	0,87237807	1,07562387	0,98644597	0,80403665	0,98425031	6,361947388	0,86323523	5,4515053	5,4515053
14	1	1,298525708	1,29852571	0,94665665	0,85600889	0,98170712	0,92777578	0,96573351	0,89216999	0,86646196	1,05817529	0,95822655	0,80351956	0,9847087	6,180164018	0,80215274	5,46950282	5,46950282
15	1	1,0379652	1,0379652	0,95324006	0,87484938	0,97154907	0,9366381	0,95514794	0,90875619	0,86912572	1,01520572	0,86184745	0,72637827	0,94251098	5,941128639	0,74963409	6,22239389	6,22239389
16	1	0,964720288	0,96472029	0,93937723	0,89745775	0,96230925	0,91721406	0,94673152	0,92166006	0,87088201	1,02656629	0,91199377	0,80745933	0,97101073	6,039373486	0,84683281	6,04880582	6,04880582
17	1	0,969298927	0,96929893	0,93689752	0,92783893	0,95699733	0,93030013	0,95217748	0,91775484	0,88904081	1,0160843	0,88391143	0,72835872	0,9707752	5,707325398	0,63381544	4,79576774	4,79576774
18	1	0,986105925	0,98610593	0,94085457	0,94226566	0,96804392	0,92402471	0,94883929	0,92884308	0,89132866	0,94458706	0,93157475	0,81278215	0,97663096	5,851730548	0,65495264	5,80809064	5,80809064
19	1	0,998342883	0,99834288	0,93536864	0,95509436	0,95443219	0,91564292	0,95153955	0,92107969	0,88305714	1,02604121	0,89592284	0,79552828	0,93722052	5,607803595	0,61972819	5,25199474	5,25199474
20	1			0,93557799	0,97239562	0,97207731	0,92995455	0,95096288	0,93211197	0,89151002	1,07205103	0,97673165	0,73731021	0,95993172	5,510286587	0,68574252	5,80450993	5,80450993
21	1			0,9372519	0,96640974	0,96694377	0,92468448	0,95473236	0,92489809	0,88972355	1,04805364	1,04227975	0,78701807	1,00065189	5,373626374	0,77593593	6,08483889	6,08483889
22	1			0,94813286	0,97019088	0,97153102	0,93411569	0,95208084	0,92332211	0,89079648	1,01326867	0,91572108	0,70424597	0,93127745	5,197016837	0,84745608	5,73764641	5,73764641
23	1										0,94510501	0,95576511	0,78247005	0,9314382	5,187098538	0,87358207	5,69568585	5,69568585
24	1										0,97540872	0,95593497	0,74634301	0,95330827	4,97649563	0,74652416	5,42276165	5,42276165
25	1										0,98141123	0,92469651	0,74753414	1,00009484	5,132018209	0,83156297	5,61048938	5,61048938
26	1										0,99523652	0,92920813	0,7675329	0,90307975	4,937131538	0,8565769	4,9470358	4,9470358
27	1										0,93938832	0,98211307	0,7755329	0,89295644	4,601390176	0,85801668	4,79416126	4,79416126
28	1										1,03864525	0,92111738	0,78313311	0,98229363	4,618806388	0,82896705	5,29049066	5,29049066
29	1										0,98696586	0,89749344	0,71253282	0,88498448	4,590307949	0,75158749	5,22931487	5,22931487
30	1										0,91665092	0,85721036	0,7027972	0,94887545	4,547628048	0,86231336	4,78170478	4,78170478
31	1										0,97217506	0,88624788	0,67468402	0,948123	4,374740615	0,85257499	4,5393322	4,5393322
32	1										0,93620045	0,88267892	0,73973381	0,88248946	4,261166106	0,7667314	4,70733671	4,70733671

DeltaTM CF intensity

		Assay 1														Assay 2														
min	background	1	2	3	4	5	6	7	8	9	10	11	12	13	14	15	16	17	18	19	20	21	22	23	24	25	26	27	28	29
0	1	1,161272535		1,31851745	1,36385213									1,29739625		2,23313535	2,52178233	1,50924818	0,81293615	0,75487251	0,89116737	0,88129813	0,79410104	0,99853204	0,90367894	1,01029834	3,66199583	3,10198826	1,92153001	3,02688885
1	1	1,175841929		1,32881819	1,35312539								1,11202768	1,30617997		2,15209855	2,48931671	1,46834354	0,81992083	0,92272135	0,85766271	0,85776106	0,73755256	1,00641735	0,85699884	1,7773352	3,51724984	3,02881785	1,94179202	2,98258666
2	1	1,200736839		1,32968373	1,35713359				1,29593231				1,12397229	1,30952324		2,08070981	2,54738644	1,52487194	0,83791459	1,24790413	0,82356618	0,84666495	0,78375477	1,04447192	1,75181054	2,11467859	3,41249663	3,05695805	1,91168808	3,02425658
3	1	1,218857212		1,33327055	1,34322655				1,28445704				1,12439612	1,30400589		2,01473203	2,54989616	1,52648329	0,78285945	1,28966517	0,85502547	0,85678854	2,39145961	1,01289426	2,91912325	2,14989018	3,3503335	2,98427605	1,87358232	3,02516314
4	1	1,210834248		1,31959365	1,31197799				1,28189421		1,27451838	1,58975385	1,11755072	1,2790689		1,89528136	2,47946202	1,49021193	0,80325245	1,26108534	0,84676937	0,83965665	2,37813808	1,00580333	2,92387773	2,1872262	3,16719161	2,96881293	1,85175815	2,98518098
5	1	1,228068661		1,31333362	1,30142012			1,45190098	1,28154376	1,54556612	1,28893357	1,53739294	1,13158032	1,27068101		1,82784704	2,47737778	1,49079003	0,84773778	1,21067376	0,87116543	0,89284213	2,32578092	1,01446281	2,85309567	2,08681188	3,02993314	2,88062807	1,76946764	3,06510944
6	1	1,223378013		1,3189586	1,29286415			1,41600205	1,27865269	1,48957532	1,27025896	1,49402858	1,126756	1,25079956		1,68985194	2,40129788	1,41226271	2,23880207	1,23999552	0,8495133	0,86808638	2,29668444	1,08503338	2,83478164	2,09178384	2,96749048	2,92084114	1,72995347	3,02513747
7	1	1,212968291		1,29204397	1,2577304			1,41413105	1,25565921	1,4562442	1,23347981	1,46134546	1,11692704	1,23189953		1,59758809	2,36242713	1,41965985	2,2229612	1,19004788	1,88998177	0,81227589	2,29916465	0,98451491	2,77345299	2,04439191	2,88572096	2,94194238	1,71435249	3,08596802
8	1	1,21739674	1,29403491	1,29147197	1,24412008	1,47090906		1,37566847	1,23619925	1,41519901	1,24178943	1,4345935	1,11090512	1,21503644		1,49960795	2,31974212	1,43509322	2,23418714	1,17010803	1,92189406	0,85245687	2,30519254	1,05850322	2,78815996	2,10585468	2,58890575	2,70443433	1,52790423	2,90669364
9	1	1,214846175	1,27685378	1,28060989	1,22127612	1,42362846	1,59149707	1,34446813	1,22261804	1,39247233	1,21993167	1,39953865	1,1130026	1,19493678		1,43376951	2,26063023	1,34709491	2,16481235	1,12844914	1,82021319	2,96428988	2,25400602	2,8917034	2,76327759	2,07767126	2,49501938	2,67446662	1,54750607	2,85695657
10	1	1,194425539	1,25357316	1,25512883	1,19880084	1,35439961	1,51270522	1,33144112	1,20473253	1,36576986	1,22358495	1,35456596	1,10586935	1,16508567		1,40064078	2,24605921	1,38377547	2,25508138	1,14904524	1,84503396	3,08373702	2,34940408	2,91016276	2,82358067	2,05264642	2,37189258	2,67786601	1,50792067	2,93164678
11	1	1,194231562	1,23250103	1,24044499	1,17788216	1,32680676	1,42721238	1,2913646	1,20039155	1,31029947	1,19381481	1,33183445	1,09764155	1,15105075		1,2382831	2,19430856	1,30687497	2,14296986	1,06848667	1,65493662	2,87170585	2,24449003	2,69054758	2,71889958	1,99584451	2,22216633	2,5740275	1,40836687	2,84674715
12	1	1,197759236	1,23604177	1,22856127	1,13070557	1,28610354	1,37633131	1,28909463	1,16920286	1,28778092	1,11612268	1,30379569	1,09694875	1,12957257		1,08525806	2,13008269	1,23079555	2,12084403	1,06102082	1,68753921	2,90903906	2,2168235	2,46660964	2,69443969	1,93800969	2,15478634	2,54690564	1,3641629	2,85611347
13	1	1,192275341	1,20405115	1,22149172	1,09786284	1,23793666	1,3129873	1,25280845	1,172326	1,25672278	1,04480794	1,26661999	1,08939312	1,11975305	1,346688731	1,02406392	2,07878844	1,24215677	2,03289046	1,07665149	1,59546595	2,94100158	2,24788628	2,34562854	2,63637771	1,91099136	1,94071524	2,45085102	1,23551348	2,77146682
14	1	1,190115333	1,20170111	1,1348466	1,06036157	1,20236616	1,25923042	1,2153332	1,14953605	1,15852313	0,98724937	1,25585621	1,07004737	1,1083966	1,315456492	0,88799142	1,97804001	1,06158894	1,89991797	0,9669338	1,43017606	2,53120464	2,03117309	2,04732757	2,5211712	1,83763488	1,86300233	2,41680062	1,22520093	2,77241379
15	1	1,174532392	1,18260162	1,1005291	1,04284122	1,18178762	1,2131694	1,20488489	1,13121545	1,13666144	0,99664989	1,20805347	1,06668867	1,09012295	1,299744203	0,86931487	1,97429482	1,06789595	1,93481167	0,94382562	1,47040477	2,71638619	2,06868424	1,80299551	2,51581725	1,86112349	1,70523084	2,31988157	1,09507621	2,69119421
16	1	1,175144281	1,15456859	1,08667599	1,02383769	1,14316952	1,17773728	1,19717834	1,13885514	1,11543135	0,97985014	1,19228433	0,98579047	1,08009316	1,245544755	0,71384638	1,88501755	1,13593111	1,84196244	0,90082854	1,50656681	2,56101885	2,0389305	1,81656355	2,53012048	1,80737364	1,49931747	2,28250935	1,01881417	2,71909312
17	1	1,15979316	1,15148326	1,07861744	1,01072303	1,10590583	1,13147505	1,14815193	1,10462188	1,08358859	0,9570145	1,15046627	0,97358927	1,06752433	1,220679327	0,69194313	1,82274071	1,09150565	1,87880261	0,81848746	1,40875765	2,55640621	2,01271924	1,70016608	2,49694171	1,84834123	1,32105983	2,12899614	0,89849484	2,49110586
18	1	1,165752894	1,14419154	1,07662772	0,99451736	1,09309409	1,11066817	1,1456452	1,09874339	1,07874166	0,94538522	1,14502123	0,97134934	1,04050611	1,20734899	0,5711579	1,77530896	0,88687939	1,99448822	0,82056582	1,35546656	2,56220127	2,00508117	1,55475175	2,47607975	1,77582569	1,20665499	2,04607302	0,86919035	2,5061296
19	1	1,150234566	1,13150685	1,0616626	0,98115969	1,07539876	1,07123494	1,00418	1,06784196	1,02905014	0,94167235	1,12156986	0,96780174	1,00757298	1,145320671	0,46898241	1,5973401	0,92676525	1,79000787	0,7305976	0,95493919	2,3387873	1,82098171	1,37448596	2,31420072	1,60600227	1,07444239	2,0326751	0,76658616	2,50106549
20	1	1,139635897	1,10685149	1,05068742	0,96498716	1,04743919	1,04957631	0,99298903	1,02217465	0,99548658	0,93333333	1,11105165	0,95427705	1,02567122	1,148443493	0,39625374	1,60606353	0,8562325	1,75446558	0,72385826	0,87969489	2,34141161	1,75243796	1,26774162	2,30684561	1,64642271	0,83672092	1,83109578	0,70126753	2,35250881
21	1	1,137982959	1,09936527	1,03558699	0,95934277	1,0278482	1,01348906	0,96709242	1,00528456	0,97816312	0,91274206	1,08801032	0,9523673	0,99442882	1,115597514	0,34252188	1,44585504	0,8208993	1,73526883	0,6158515	0,98285367	2,18764858	1,83329118	1,10859339	2,24682616	1,56866117	0,70515464	1,73481364	0,67835052	2,19587629
22	1	1,138057032	1,09931368	0,98509425	0,94480425	1,00125665	0,99159357	0,96717836	0,9934576	0,9752924	0,90800439	1,06871345	0,95105994	0,97686404	1,097368421	0,23402062	1,29103093	0,69979381	1,60876289	0,55268041	1,05329897	2,04391753	1,61195876	0,89175258	2,13865979	1,35226804	0,74801061	1,69018568	0,56339523	2,20671972
23	1	1,117053718	1,07064665	0,96215498	0,93994839	0,98064743	0,97367409	0,95117706	0,98723092	0,94634057	0,89753595	1,0415682	0,93483558	0,96935055	1,062709536	0,23140207	1,36063948	0,68207013	1,58177257	0,51239862	0,95583173	2,18939261	1,71188517	0,84789078	2,2225334	1,50312154	0,65936142	1,6493482	0,51048555	2,22350274
24	1	1,108589824	1,07113693	0,93333857	0,92144315	0,93039416	0,94423558	0,9365682	0,97120104	0,93304936	0,87396981	0,91182517	0,93227151	0,95949625	1,063024354	0,18081312	1,22681883	0,70827389	1,44079886	0,52757965	0,80527817	1,93319068	1,52472658	0,77330005	1,98561579	1,38742273	0,48424894	1,41067998	0,43219362	1,96542451
25	1	1,114079221	1,06069318	0,93012298	0,9196614	0,90472768										0,12244898	1,13753865	0,62560297	1,46839827	0,460235	0,79579468	1,65132962	1,55275201	0,58676562	1,93605442	1,26580087	0,44312252	1,37591317	0,41327489	1,94802755
26	1	1,092412295	1,04634696	0,91559382	0,90988091	0,88421307										0,11785544	0,94924722	0,55071871	1,3521357	0,39702977	0,76381225	1,64616118	1,43334015	0,52006267	1,87151713	1,17841815	0,36719624	1,21839338	0,36137838	1,77265608
27	1	1,093048172	1,02981665	0,90848814	0,89854252	0,87319331										0,08357908	0,96943106	0,50766022	1,21570884	0,4282529	0,65036323	1,54801122	1,30144573	0,49715889	1,79529598	1,12968424	0,28736323	1,19826861	0,29986774	1,76529999
28	1	1,08874757	1,02811082	0,89675146	0,89095917	0,86062054										0,06477607	0,89958159	0,42538354	1,14706338	0,32589493	0,62157136	1,49511855	1,23911359	0,29521153	1,70215404	1,06772044	0,26654305	0,96256808	0,28346274	1,63286592
29	1															0,05589383	0,72974239	0,41030445	1,01545667	0,25839188	0,5587822	1,27088212	1,18110851	0,2295082	1,58891491	0,90897736	0,18025527	0,93149258	0,21437874	1,55352956
30	1															0,04293263	0,72275429	0,39745707	1,06439894	0,26898943	0,45426024	1,20310436	1,12929326	0,24488111	1,5582893	0,83404888	0,14182344	0,85594001	0,18839626	1,36955664
31	1															0,03691425	0,61912015	0,40731117	0,95027327	0,26986829	0,37039692	1,17229639	1,16584535	0,17901622	1,44485261	0,78828062	0,12285867	0,75856531	0,17157388	1,25401499
32	1															0,02173718	0,52762059	0,2899488	0,80265876	0,21270098	0,38767628	0,86697207	0,85295967	0,15144166	1,26093596	0,74804635	0,10412079	0,677257	0,17143756	1,19440075
33	1															0,02498107	0,46498864	0,26778955	0,84632854	0,26135503	0,32475397	0,79806964	0,82305072	0,08894777	1,24394398	0,57361847	0,11013645	0,6517219	0,11533463	1,14132554
34	1																										0,06524259	0,58618977	0,09107468	0,95148203
35	1																										0,06909399	0,41558002	0,0765453	0,91515665
36	1																										0,05052328	0,40562974	0,06964995	0,79646337

Bleaching CF intensity

		Assay 1												Assay 2						
min	background	1	2	3	4	5	6	7	8	9	10	11	12	13	14	15	16	17	18	19	20
0	1														1,44546165	2,58592033	1,32476974	2,33969564	0,8757422	1,33491514	0,70607555
1	1								1,38046496			0,56235262			1,858644318	2,77909972	1,39879034	2,42894478	0,93700297	1,39526838	0,76508656
2	1								1,40609038			0,59612668			1,965280646	3,23313786	1,42220779	2,49467377	0,96843741	1,42835858	0,83408786
3	1		2,68033376						1,48208819			0,60737797			2,097753653	3,26548312	1,53339577	2,47003814	1,01089892	1,50587022	0,87591786
4	1		2,60883467						1,49299109			0,62761344			2,170492788	2,72040634	1,51270499	2,48421671	1,03929031	1,52143617	0,91418537
5	1		2,5994059			2,72598671	2,34129019		1,54004393			0,62315068			2,299083687	1,99413613	1,5666851	2,48722463	1,09302156	1,60666996	0,97426011
6	1		2,45874777	1,11027378		2,22058612	2,33895941		1,56139128	1,51608868	1,51748323	0,62761223			2,367404278	1,58261615	1,63774556	2,52107889	1,18726224	1,67486748	1,06092922
7	1	1,245204356	2,33225884	2,19569621		2,30239801	2,47060745		1,56160565	1,61089452	1,68734345	0,58778559	2,24350402		2,367847886	2,3278217	1,60985261	2,51047634	1,23569187	1,68565413	1,07010449
8	1	1,193629231	2,30497716	2,16582738		2,23503923	2,42637311		1,5829254	1,8354374	1,63405551	0,56037007	2,20002342		2,444290901	2,4710289	1,62708831	2,47480774	1,28301512	1,74146447	1,12647507
9	1	1,456605756	2,23016022	1,59218541		2,37977312	2,72730229		1,57967806	1,83332708	1,57475017	0,60105312	2,11439221		2,488837192	3,22805978	1,65955296	2,49902836	1,34176289	1,78224914	1,17629733
10	1	1,103780941	2,11827829	1,69625606		2,39569705	2,62633311		1,56061267	1,94764038	1,79933658	0,57333915	1,97555141		2,518151423	3,23117993	1,63904797	2,46492122	1,3512207	1,79900612	1,2198162
11	1	1,09636151	1,99042575	1,51259371	1,76988789	2,24937066	2,62857143		1,49295	1,92241557	1,65323612	0,54014719	1,79442878		2,467790082	3,31555235	1,65154461	2,47488879	1,43528943	1,83490624	1,29876412
12	1	1,086791244	1,92405948	1,44450109	2,14582211	1,98153192	2,60402861	2,60402861	1,51921817	1,89522131	1,62507464	0,52546644	1,56929172		2,527010297	3,36770803	1,6732239	2,50303218	1,45362813	1,88324858	1,34876565
13	1	1,036393918	1,81392838	1,37797243	2,43873176	1,81195448	2,60379977	2,60379977	1,51839743	1,81047405	1,59864911	0,50618388	1,50858958		2,596397619	3,30754809	1,63474586	2,46701401	1,47684404	1,87112211	1,38239712
14	1	1,016654763	1,72878385	1,2020648	2,54548406	1,77680021	2,48574768	2,48574768	1,51240179	1,74917213	1,70193494	0,47230699	1,55071099		2,65645088	3,19653847	1,62114306	2,38312084	1,50448445	1,834107	1,37163141
15	1	1,000510917	1,62062741	1,20031336	2,66531558	1,8047958	2,38073504	2,38073504	1,52256548	1,74219149	1,72962294	0,45842842	1,45120747	1,26267925	2,60506148	3,20025731	1,5809501	2,34929184	1,47185364	1,86366759	1,42568989
16	1	1,00261192	1,57259493	1,0612066	2,74109116	1,77548448	2,52318764	2,52318764	1,52167163	1,71312718	1,6447789	0,45370692	1,37715757	1,36532174	2,708579152	3,16537974	1,63153164	2,36968999	1,54611041	1,88318543	1,47371211
17	1	0,903115616	1,51411411	1,23430931	2,61073574	1,78138138	2,37263514	2,37263514	1,46914414	1,6628003	1,51655405	0,39793544	1,26876877	1,31415165	2,691441441	2,66973875	1,56858436	2,295204	1,52718207	1,85485567	1,48901729
18	1	0,882882519	1,39031086	1,23924102	2,52507065	1,65571256	2,38046023	2,38046023	1,51570448	1,58873637	1,61788454	0,39386354	1,22240614	1,34610416	2,643076302	2,93677974	1,60690448	2,3373247	1,58733095	1,90745694	1,57585681
19	1	0,844048644	1,31977904	1,17044381	2,87719225	1,56064775	2,35634622	2,35634622	1,48654723	1,56720086	1,4656697	0,34865892	1,1212955	1,33948037	2,694419357						
20	1	0,804983772	1,22590342	1,20834695	3,07521401	1,56365854	2,21407567	2,21407567	1,48001481	1,51172973	1,4326167	0,32594916	1,05700407	1,24550742	2,656341894						
21	1	0,770245813	1,12922957	1,11727108	3,07649309	1,61178438	2,17566608	2,17566608	1,49691874	1,41256787	1,43456049	0,3205883	0,94673662	1,19314563	2,713614808						
22	1	0,723481887	1,03508897	1,0464141	3,11171133	1,50107802	2,21100765	2,21100765	1,39564527	1,41019262	1,32864217	0,26379511	0,86208449	1,13947923	2,713057076						
23	1	0,637340077	0,97248441	1,09065872	3,41253349	1,33827395	2,12323177	2,12323177	1,39445682	1,34838887	1,29946671	0,25087003	0,80033049	1,03402519	2,680003004						
24	1	0,610362336	0,87508307	1,0668049	3,08147912	1,38931866	2,14711434	2,14711434	1,40697025	1,32663423	1,1999362	0,24547412	0,71579339	1,03602095	2,706489087						
25	1	0,551503625	0,79164255	0,9823865	3,71978279	1,32798589	2,05612062	2,05612062	1,37848342	1,26039858	1,15940351	0,21367733	0,66153973	0,93767744	2,695719287						
26	1	0,491206725	0,6850459	0,95348966	3,83978542	1,01415773	1,85145449	1,85145449	1,30809645	1,17669506	1,02715408	0,19975666	0,57709324	0,84111271	2,630461232						
27	1	0,434201619	0,6258131	0,92126564	4,20503311	1,06849154	1,90039735	1,90039735	1,36388521	1,0799117	1,00986019	0,18772627	0,51255335	0,88835909	2,630110375						
28	1	0,365403187	0,54333655	0,83637132	4,16205939	0,98503139	1,84620956	1,84620956	1,27915258	1,06409947	0,90379044	0,15149686	0,4884114	0,79810478	2,578947368						
29	1	0,375362144	0,49082569	0,70346451	4,15300579	0,88978754	1,67268228	1,67268228	1,22489136	0,95473201	0,84005311	0,13562289	0,3799493	0,67865765	2,419664413						
30	1	0,301001931	0,40348865	0,61528247	4,01780541	0,76237325	1,53730082	1,53730082	1,11371318	0,86329068	0,73756639	0,11443747	0,34307098	0,61280782	2,334862385						
31	1	0,238290681	0,31808305	0,54901014	4,05166586	0,6785973	1,40246258	1,40246258	1,06597055	0,74088605	0,66556012	0,09107919	0,26237325	0,55070014	2,245714631						
32	1	0,184693385	0,26273539	0,45159343	4,07598986	0,5697127	1,37361178	1,37361178	0,96257846	0,68306374	0,54973443	0,08715596	0,24788749	0,45980203	2,158981169						
33	1	0,180226944	0,20907774	0,3816393	3,97139063	0,55003621	1,26750362	1,26750362	0,86069532	0,60290922	0,52009899	0,074662	0,21330275	0,42322549	2,060417673						

d)

Full-length CF intensity

		Assay 1									Assay 2							
	Background	1	2	3	4	5	6	7	8	9	10	11	12	13	14	15	16	17
0	1			0,34990896	0,38696106	0,3582174	0,3526313	0,35178278	0,34651488		0,23511669	0,48403273	0,11506994	0,26610866	0,253365004	0,22516307		
1	1	0,458306427	0,45830643	0,32903687	0,36236632	0,33815091	0,33904901	0,32946929	0,33615514		0,30179118	0,42509765	0,10050322	0,30749199	0,668402717	0,09796953	1,3767815	
2	1	0,407737076	0,40773708	0,31249203	0,3545785	0,32591506	0,33548017	0,32126004	0,31679633		0,21065635	0,44113207	0,21027204	0,15421779	0,433665486	0,14126108	0,14933158	
3	1	0,38427932	0,38427932	0,30981211	0,34677182	0,31253383	0,33019408	0,30517281	0,30746153	0,31605969	0,28486521	0,61382934	0,08039694	0,1904585	0,572118446	0,04977712	0,04309064	
4	1	0,359248093	0,35924809	0,29884678	0,54153403	0,31506397	0,30719563	0,31206078	0,30812661	0,31476365	0,30247926	0,8582998	0,14378695	0,14805982	0,629900134	0,15824514	0,27659363	
5	1	0,334603087	0,33460309	0,29369863	0,50811824	0,29364095	0,58249459	0,30085076	0,29704398	0,29736121	0,32546536	1,14047208	0,10511418	0,17405488	0,967952408	0,11398964	0,37895797	
6	1	0,329695594	0,32969559	0,28123983	0,47193296	0,28178219	0,75732184	0,49720686	0,28641935	0,28351774	0,54133157	1,24069373	0,12560531	0,11779764	1,030825351	0,10636346	0,71578813	
7	1	0,310870983	0,31087098	0,28789967	0,4904355	0,2966097	0,84536935	0,71143881	0,2969129	0,28577729	0,64512954	1,1888064	0,15750626	0,20671359	0,977343232	0,12233371	0,56699686	0,76194348
8	1	0,30711857	0,30711857	0,27222507	0,48458843	0,70267291	0,84402105	0,76450288	0,26765868	0,27710638	0,87699303	1,22905931	0,1585531	0,70292008	0,880549662	0,16497929	0,46882894	1,90987168
9	1	0,290460103	0,2904601	0,26897902	0,46271195	0,81157553	0,83010359	0,80082977	0,2639798	0,27807556	0,77750166	1,08663568	0,15077302	0,84655221	1,143317841	0,0557716	0,73189794	1,87290145
10	1	0,283254334	0,28325433	0,27214454	0,43865858	0,83266922	0,87570845	0,85501794	0,25727014	0,26365549	0,70425854	0,89143242	0,24009426	0,77774785	0,643359704	0,11681535	0,37007238	1,53129103
11	1	0,278661035	0,27866104	0,44521044	0,44006494	0,87723242	0,80357187	0,90323467	0,25912327	0,26694143	0,84668435	1,10401667	0,47093596	1,1955665	1,043652899	0,14907162	0,51743085	2,90568397
12	1	0,279712984	0,27971298	0,60315951	0,43589556	0,88047003	0,79221824	0,909676	0,25624711	0,26158609	0,89343045	1,26516223	0,8098928	0,64442814	0,818912293	0,15104064	0,48934304	2,38558313
13	1	0,275365357	0,27536536	0,69376022	0,43421053	0,9271144	0,8298855	0,87727316	0,2476667	0,41256134	0,9470927	1,14323366	0,95480428	0,77747047	0,847330231	0,05879673	0,35349624	1,7243354
14	1	0,276434369	0,27643437	0,73422269	0,41939501	0,91787416	0,84101741	0,91962254	0,46432592	0,54882284	0,99586922	1,24175593	1,0499895	0,71361059	0,966743681	0,14912833	0,56651264	2,55187986
15	1	0,393152941	0,39315294	0,75559285	0,53246983	0,92132787	0,85387097	0,89646289	0,62266448	0,61878819	0,92169452	1,12661984	0,93738579	0,87844294	0,985364872	0,06816315	0,53870011	1,60604181
16	1	0,581843111	0,58184311	0,79602743	0,64303927	0,91108291	0,86954092	0,91285264	0,68576884	0,70702884	0,95488011	1,04922007	0,86014796	0,97641501	1,032748017	0,17195829	0,51833497	1,80408236
17	1	0,736313362	0,73631336	0,80105432	0,71661701	0,89692872	0,82986477	0,94292918	0,75677286	0,73149209	1,24441976	1,11820044	1,13580033	0,97931058	1,046967751	0,10136425	0,40882424	2,25830527
18	1	0,79434282	0,79434282	0,83417074	0,79809141	0,9769244	0,86206381	0,93390055	0,78592699	0,7788215	1,2485436	1,15299064	0,89711287	0,87974836	0,992601627	0,08390172	0,50482258	2,32125949
19	1	0,884903921	0,88490392	0,83495828	0,82026574	0,94277163	0,83008344	0,92225195	0,81001723	0,76621168	1,04631446	1,30903167	1,04519339	0,97340947	1,033036715	0,12230241	0,36550589	2,72060678
20	1			0,82232326	0,84692877	0,94971918	0,8369885	0,92912543	0,79339396	0,76626995	1,17469644	0,95913488	0,92859933	0,8835305	0,975105874	0,12501758	0,44156209	1,18141614
21	1			0,85777807	0,86536324	0,91019857	0,85272129	0,91643235	0,84313084	0,76270189	0,84852641	1,12737871	0,94397082	0,7889282	0,918909652	0,10772564	0,82187554	1,74555429
22	1			0,88587581	0,90115714	0,95349351	0,90190796	0,95053441	0,80207137	0,7944307	0,88034752	1,19092591	1,08644518	0,87352586	1,114117931	0,07564959	0,89923578	1,39824632
23	1										1,17129196	1,24431081	1,10405679	0,94825915	1,061763691	0,09408324	0,72203874	1,33280755
24	1										0,97321373	1,15243711	0,93253246	0,85916818	1,187503921	0,14839094	1,1726366	0,89966125
25	1										0,90814478	0,97117812	1,01172389	0,80626926	0,827889545	0,11562824	1,05546005	1,34928526
26	1										1,14173553	1,23781447	0,99353873	0,73352987	1,07183772	0,18146547	1,07898636	1,99282081
27	1										1,10055266	1,3236684	0,98252303	0,90745695	0,827585102	0,19700441	0,85709251	1,18462155
28	1										0,9771853	0,97011304	1,02707581	0,89646091	0,913446174	0,09333018	1,04249275	1,18887968
29	1										1,30405563	1,09540922	0,97913847	0,83561239	1,080039203	0,17762636	1,13744341	1,10850796
30	1										0,9600781	1,14967385	0,90549795	0,96768086	0,894108598	0,13037112	0,66170663	0,93164909
31	1										0,99705727	1,01159437	1,03448885	0,78988876	0,906450474	0,1469013	0,93911483	0,64360544
32	1										1,18258731	1,18069076	0,89128921	0,80348491	1,122431139	0,08623372	0,8460091	1,04064246

DeltaTM CF intensity

		Assay 1													
min	background	1	2	3	4	5	6	7	8	9	10	11	12	13	14
0	1	0,457852993		0,4564235	0,46366319									1,04466085	
1	1	0,456109455		0,44193082	0,45163912								0,62253901	1,00486702	
2	1	0,430918532		0,42240572	0,42478931				1,09924024				0,57588786	0,97432122	
3	1	0,415665313		0,42185231	0,42521939				0,99853809				0,55968513	0,98290694	
4	1	0,404493487		0,39644664	0,39716369				1,00017041		0,5776768	0,56353309	0,57279182	1,06316387	
5	1	0,390413107		0,39727209	0,39202007			0,52112751	0,52736186	0,53226408	0,54260137	0,53173123	0,53162466	1,03543454	
6	1	0,380590655		0,36569459	0,36999296			0,53354649	0,50910216	0,51260894	0,50544067	0,50281058	0,51064927	1,05842917	
7	1	0,362319097		0,35055044	0,36104681			0,51288627	0,52499038	0,50375689	0,50026927	0,50673163	0,51919477	1,09049878	
8	1	0,363700391	0,39159066	0,35493186	0,36158749	0,43159489		0,48755141	0,5045928	0,47710986	0,49395424	0,50528234	0,49311695	0,92043244	
9	1	0,345888721	0,38612496	0,33969329	0,34289313	0,39133321	0,47942609	0,47717076	0,49271816	0,47707479	0,48710382	0,48849541	0,47841839	1,04868159	
10	1	0,329951134	0,37037518	0,32823027	0,33073039	0,37894703	0,47527222	0,48616108	0,49090996	0,48515374	0,47522425	0,48102845	0,48496187	1,0172207	
11	1	0,33956629	0,36774445	0,33148292	0,33297624	0,35456434	0,47007231	0,46923256	0,48635409	0,46204805	0,47347796	0,4715652	0,47231164	1,03890833	
12	1	0,320835648	0,35780411	0,30929458	0,31599087	0,35515028	0,47236134	0,47110345	0,47324652	0,45312027	0,46998532	0,47059098	0,48084046	0,49789187	
13	1	0,319897849	0,3423587	0,30743669	0,32263623	0,3614658	0,46498935	0,43521477	0,45451722	0,46046326	0,45132233	0,44732872	0,44315761	0,48974973	0,489749734
14	1	0,320264148	0,35331337	0,31802196	0,31759195	0,35629271	0,46364627	0,43918062	0,44549721	0,44901137	0,44193857	0,45688486	0,44291719	0,48740019	0,487400191
15	1	0,308515757	0,33984845	0,30307313	0,3154318	0,33744287	0,43834783	0,43973913	0,45265217	0,44586957	0,42695652	0,43665217	0,43756522	0,47086957	0,470869565
16	1	0,298730048	0,33618781	0,29951649	0,30210882	0,34023651	0,45790129	0,4394333	0,44890742	0,4469864	0,43938964	0,45139601	0,44716104	0,46554171	0,465541706
17	1	0,304069567	0,34516703	0,29479968	0,29689473	0,33233842	0,4393378	0,4242606	0,43449768	0,43316985	0,42486026	0,43214186	0,42271861	0,48756773	0,48756773
18	1	0,303862132	0,34191096	0,30075011	0,30788427	0,32729034	0,42503826	0,41155548	0,4166839	0,41300302	0,42528641	0,41689069	0,41196906	0,43988585	0,439885851
19	1	0,296447459	0,32783398	0,29633249	0,30317314	0,33237526	0,4448278	0,41353277	0,43638504	0,41747001	0,40573945	0,41328923	0,4117468	0,44113409	0,44113409
20	1	0,297616154	0,33548313	0,2938123	0,30150581	0,34186103	0,43170597	0,41724202	0,43604925	0,42703489	0,41990535	0,42338817	0,42240479	0,45948659	0,459486591
21	1	0,288820865	0,3255814	0,28574595	0,299126	0,35126113	0,4326173	0,4074163	0,40817633	0,4426177	0,39681587	0,41869675	0,42197688	0,44701788	0,447017881
22	1	0,290004314	0,32665841	0,29562628	0,29134023	0,32980337	0,41752761	0,40205043	0,40739421	0,427186	0,40533587	0,40668171	0,41812136	0,42235681	0,422356806
23	1	0,292343451	0,31726471	0,30664705	0,29399387	0,31987787	0,41237394	0,3851575	0,41595608	0,40139392	0,39150411	0,40688393	0,40984309	0,41778608	0,417786084
24	1	0,288521969	0,32771928	0,29023099	0,29414521	0,33474833	0,40984479	0,41051531	0,4164711	0,42140138	0,60054825	0,41394679	0,41023922	0,42948705	0,429487053
25	1	0,281217576	0,31247895	0,30288494	0,28030129	0,32686996									
26	1	0,282174097	0,32805229	0,3007055	0,28905173	0,32968982									
27	1	0,277578968	0,30563917	0,28122214	0,27915509	0,32589626									
28	1	0,265627211	0,3027543	0,28749694	0,27329448	0,33038735									
29	1														
30	1														
31	1														
32	1														
33	1														
34	1														
35	1														
36	1														

	Assay 2														
min	15	16	17	18	19	20	21	22	23	24	25	26	27	28	29
0	0,24059544	0,25485478	0,22051598	1,11789132	0,77593598	0,95371873	1,15216296	1,09259612	1,00616711	0,89724103	0,97818568	0,21179873	0,2178161	0,20435675	0,21345722
1	0,24365808	0,24910545	0,23774073	0,9083879	0,85624259	0,98689425	1,09139848	0,94194803	1,15119148	0,86532156	0,65671897	0,2036492	0,20709051	0,16907879	0,17955997
2	0,22334636	0,21704412	0,2440671	0,96652516	0,19882932	0,9471135	1,01041647	0,76483459	0,97580087	0,69594001	0,20875956	0,19226773	0,26472953	0,19170755	0,19331581
3	0,226508	0,2324818	0,21885467	0,92179672	0,19755513	0,83766736	1,01880887	0,20585737	0,96481568	0,2156292	0,24504013	0,19565561	0,2270031	0,1708638	0,19016481
4	0,25084502	0,21522268	0,17714846	0,81286888	0,21273577	0,73812151	0,81689697	0,15494142	1,13017741	0,21434701	0,2166763	0,2247899	0,2067707	0,18200862	0,18464358
5	0,17419392	0,24695598	0,16258208	0,72547817	0,199004	0,8736027	1,10574132	0,16992534	0,99323733	0,20848973	0,20768834	0,23462665	0,21347401	0,18048334	0,17790912
6	0,19384092	0,19032807	0,20212009	0,21587744	0,19740019	0,79650263	0,79323739	0,17103064	1,00646859	0,17712782	0,19877747	0,20731251	0,20116212	0,19101906	0,20977607
7	0,19241739	0,17973341	0,1578143	0,16873299	0,20804354	0,20114581	0,98090047	0,22262359	1,15380152	0,18986752	0,1855687	0,19877731	0,20885094	0,15495927	0,1757239
8	0,17857878	0,19436134	0,23585189	0,17910117	0,14495576	0,23059632	0,93956087	0,17113866	1,01036868	0,21904038	0,18492663	0,22992001	0,25185572	0,18657045	0,20039049
9	0,17462999	0,20117695	0,20022058	0,20996938	0,15872649	0,18405485	0,5479843	0,19307866	0,32715549	0,18221924	0,19155156	0,21621847	0,20011194	0,19473196	0,19476538
10	0,18632776	0,19819644	0,19702608	0,20307295	0,18142124	0,19262972	0,45785193	0,19851154	0,2312817	0,17094799	0,14767578	0,23114249	0,27775604	0,16621535	0,1763113
11	0,16304048	0,19947061	0,17531136	0,25810153	0,20695248	0,17783592	0,40916185	0,19232535	0,2302855	0,20530004	0,1635148	0,22446333	0,18920967	0,15853214	0,15920253
12	0,16726268	0,24862699	0,18324887	0,19688028	0,22585434	0,18320499	0,28522118	0,20248203	0,2522396	0,17621377	0,16379632	0,2077178	0,21445707	0,18880997	0,18042929
13	0,19367224	0,21258706	0,22333175	0,23589202	0,18478778	0,23675516	0,33146318	0,20990833	0,29451158	0,19182689	0,19273469	0,18830746	0,20384129	0,19223308	0,18114625
14	0,18029203	0,2224367	0,1959537	0,21215644	0,15331184	0,17980946	0,21173236	0,20374798	0,26987504	0,23884417	0,20345551	0,21790697	0,2265586	0,17826159	0,1847107
15	0,14475704	0,2255938	0,14270164	0,21726402	0,14688005	0,16382359	0,27238122	0,20982671	0,50757591	0,18234924	0,1565215	0,21018016	0,20118326	0,17386592	0,18889046
16	0,1782487	0,19727134	0,17868812	0,17694461	0,18659768	0,2013537	0,3790354	0,18174988	0,16367696	0,16091286	0,18359261	0,19561883	0,19955593	0,205721	0,1741174
17	0,17894471	0,22838424	0,18809186	0,28368804	0,16564486	0,24145962	0,25193956	0,18298517	0,1741186	0,16913818	0,15905105	0,20981358	0,21311077	0,14997829	0,15401147
18	0,16660731	0,20030434	0,15483537	0,23433807	0,15997669	0,24156441	0,34432609	0,15907016	0,13204261	0,17385955	0,18552789	0,19206574	0,22804454	0,15970178	0,15130014
19	0,17629379	0,25388692	0,2389281	0,18941717	0,15515567	0,80308048	0,28531879	0,2389434	0,17488662	0,1853945	0,20977524	0,17721994	0,18055714	0,14868623	0,1585772
20	0,16582242	0,1985493	0,17674906	0,15204477	0,14408847	0,55454039	0,41736063	0,13405028	0,26623084	0,16445659	0,15993476	0,17321848	0,21689402	0,15999481	0,14903271
21	0,15614363	0,20055364	0,16185216	0,19659342	0,17280566	0,36163759	0,26170514	0,15187878	0,15075297	0,19477888	0,15463371	0,18008351	0,1956023	0,17298676	0,15999031
22	0,1609984	0,21448211	0,19494127	0,1829685	0,13491725	0,16907368	0,30226909	0,15411105	0,20065403	0,18251468	0,17536038	0,17852948	0,20499569	0,16033485	0,15585026
23	0,16351615	0,16762579	0,18284158	0,1951075	0,13210128	0,13071459	0,27285047	0,15648184	0,19743966	0,1730591	0,18329541	0,16525993	0,15657585	0,13276023	0,14194411
24	0,15995654	0,19436734	0,14943587	0,21356689	0,13124333	0,1607648	0,32009858	0,18209764	0,2015887	0,20117794	0,21493166	0,18658235	0,17650944	0,15228108	0,14607723
25	0,17552352	0,14862682	0,19628738	0,15697869	0,12663151	0,19886	0,3114945	0,15549121	0,14879751	0,16285549	0,14965099	0,1634855	0,16392291	0,13367531	0,14165474
26	0,13072133	0,17681773	0,17676761	0,15758479	0,15510393	0,29008533	0,32549398	0,13721166	0,18360878	0,15648219	0,15857463	0,19157266	0,17697952	0,15619866	0,16912167
27	0,15509131	0,1547627	0,16386639	0,20892234	0,14979705	0,13504616	0,27264816	0,15399595	0,1566735	0,14430806	0,15263283	0,1694854	0,16206991	0,16132033	0,15383792
28	0,15286797	0,14561726	0,19424252	0,22992977	0,19024171	0,13421803	0,34164476	0,18035663	0,1785101	0,17743911	0,17783304	0,16997563	0,14758424	0,1710209	0,14517886
29	0,16162297	0,18729674	0,14496044	0,19407292	0,15189035	0,14827166	0,25663131	0,11610553	0,17518714	0,14218138	0,17095351	0,19813067	0,18805577	0,15069582	0,15947461
30	0,13984906	0,17132936	0,152611	0,19063068	0,23693866	0,23645707	0,28926641	0,22345434	0,2423121	0,15302922	0,15392902	0,1632446	0,15938132	0,17060986	0,16263315
31	0,16200066	0,20399794	0,17283195	0,17257158	0,16175331	0,18337684	0,36088239	0,19320571	0,16529431	0,15542638	0,15993074	0,1636231	0,22571448	0,13845236	0,15815927
32	0,1438628	0,1751293	0,15229062	0,18607565	0,16579235	0,15904763	0,30831603	0,1633844	0,23422239	0,1604236	0,16829859	0,15085498	0,15273737	0,16929719	0,15361933
33	0,18964617	0,15302621	0,17671214	0,25813832	0,18098381	0,2153423	0,37553644	0,15224593	0,15795912	0,18164505	0,15850135	0,19149068	0,19072048	0,15075266	0,16168674
34												0,15274729	0,20095648	0,16850507	0,15360055
35												0,17069639	0,23814988	0,14722837	0,1662432
36												0,16115409	0,20989896	0,16454847	0,14953689

Bleaching CF intensity

min	background	3	4	5	6	7	8	9	10	11	12	13	14	15	16
0	1														0,337164654
1	1								0,26906912			0,19144814			0,261446765
2	1								0,22882007			0,18267283			0,191375336
3	1		0,25089761						0,23253271			0,20894722			0,191036276
4	1		0,25312829						0,22702992			0,18560893			0,218841839
5	1		0,25485062			0,26515007	0,29584034		0,21765818			0,18294523			0,178020911
6	1		0,17714813	1,04440323		0,43835156	0,29865124		0,22216794	0,45548016	0,48393622	0,13438771			0,134708302
7	1	0,442042121	0,25337097	0,21937097		0,47996774	0,20870968		0,23835484	0,38309677	0,40375806	0,15230645	0,19079032		0,161516129
8	1	0,341057168	0,16482853	0,17663631		0,36869117	0,26320732		0,18873529	0,33798511	0,43265122	0,17488917	0,18321722		0,169298303
9	1	0,342500856	0,2373651	0,81459347		0,3462402	0,1935947		0,1866335	0,37880925	0,54117038	0,17369603	0,2911516		0,170624421
10	1	0,42700303	0,19129025	0,49269255		0,1808752	0,18879572		0,18508914	0,15659221	0,32429004	0,14072299	0,17656261		0,143457121
11	1	0,304803248	0,18029217	0,62923729		0,3438426	0,16627652		0,19374481	0,17054344	0,31650764	0,12931137	0,27669788		0,171550907
12	1	0,268564772	0,18780953	0,66912506	0,47824254	0,42563014	0,14703171	0,14703171	0,20255987	0,1559934	0,36937304	0,14688878	0,36121176		0,150833637
13	1	0,304549626	0,147926	0,5993776	0,30550716	0,44235501	0,18105409	0,18105409	0,19831633	0,15083851	0,41864269	0,13031798	0,40406687		0,145984334
14	1	0,279089237	0,19079904	0,67056902	0,25696522	0,42172875	0,17756855	0,17756855	0,20843512	0,18036837	0,25288902	0,17265516	0,18728555		0,153029014
15	1	0,409959964	0,20204269	0,67235795	0,28100082	0,3376275	0,21679621	0,21679621	0,22364989	0,20260601	0,18275582	0,13569211	0,1994273	0,60863617	0,152336756
16	1	0,265383275	0,21695934	0,94970517	0,23888108	0,36238597	0,12910468	0,12910468	0,15203282	0,14605886	0,18217359	0,13826562	0,15310375	0,2617189	0,12924661
17	1	0,312459165	0,17935971	0,6835781	0,35836434	0,32588132	0,16535523	0,16535523	0,18384797	0,18028293	0,31369486	0,14402182	0,16714485	0,2712979	0,15064057
18	1	0,280254685	0,19111482	0,72959852	0,45285873	0,45149023	0,18732624	0,18732624	0,177401	0,21002895	0,19692015	0,14756767	0,19269941	0,17508175	0,134991861
19	1	0,274207672	0,17254675	0,69145811	0,31396531	0,44476301	0,18371843	0,18371843	0,21373086	0,18710807	0,17743348	0,16821084	0,20548274	0,16403028	0,131080165
20	1	0,278993331	0,15682679	0,61049315	0,2649907	0,3137894	0,209537	0,209537	0,17611267	0,16076546	0,15663665	0,13811134	0,17236415	0,16117291	0,176520121
21	1	0,319820121	0,15505173	0,60448091	0,27323099	0,30163149	0,15668456	0,15668456	0,20261788	0,16049895	0,16139567	0,13405249	0,22680247	0,17305299	0,149015619
22	1	0,235688607	0,19327353	0,90282566	0,25137251	0,18252192	0,16409636	0,16409636	0,14638309	0,21602661	0,20093405	0,145738	0,19446964	0,18568021	0,137687733
23	1	0,171427836	0,16675464	0,59940523	0,31127618	0,16797765	0,1668705	0,1668705	0,18582077	0,15489785	0,1693809	0,11559406	0,16075544	0,18099309	0,129047208
24	1	0,144950726	0,14441735	0,55183887	0,31225236	0,16885096	0,17318145	0,17318145	0,16780961	0,15824698	0,12682871	0,13040994	0,17107335	0,17709286	0,131540181
25	1	0,179339303	0,1488072	0,42837117	0,21263658	0,17121021	0,14195828	0,14195828	0,1830518	0,18129797	0,15862612	0,12043859	0,20286887	0,17522995	0,168099393
26	1	0,14671034	0,15068678	0,50157036	0,23992074	0,20395757	0,20147735	0,20147735	0,17774004	0,18540984	0,17191691	0,13674903	0,18247132	0,14707429	0,155579819
27	1	0,152770071	0,17970293	0,46326575	0,1775994	0,2260837	0,19409206	0,19409206	0,14880822	0,16530088	0,14030379	0,13427712	0,16231981	0,15052459	0,159712992
28	1	0,153310846	0,1531231	0,45352425	0,15756635	0,17709161	0,16039501	0,16039501	0,14550074	0,16329877	0,17555212	0,11200741	0,17536438	0,15605189	0,168079953
29	1	0,212187017	0,15204671	0,26193888	0,1764658	0,15716583	0,15735358	0,15735358	0,16807995	0,15859268	0,14704023	0,13253397	0,16065785	0,15388659	0,127189551
30	1	0,123584888	0,15485034	0,19769327	0,12566258	0,15856765	0,19357544	0,19357544	0,17257327	0,14023142	0,15749125	0,15587667	0,16446277	0,12862891	0,140531813
31	1	0,182148154	0,16546407	0,18354997	0,15038206	0,1743005	0,20581627	0,20581627	0,16470058	0,16558923	0,16125863	0,13020595	0,1928495	0,14195866	0,139104967
32	1	0,160820562	0,1729112	0,17836826	0,17064577	0,16777956	0,15686544	0,15686544	0,16710369	0,17172217	0,16945673	0,12648864	0,16011965	0,16244767	0,156852928
33	1	0,152184389	0,16150895	0,15094529	0,1430726	0,15498802	0,13770315	0,13770315	0,16880589	0,15670273	0,15610196	0,11931687	0,18446365	0,152272	0,129930598

g)

		DOPS outer leaflet LactC2		DOPS inner leaflet LactC2		DOPS inner leaflet LactC2+LIPP	
		0	30	0	30	0	30
Assay 1	1		1,832337213		2,48		1,466491459
	2		1,637889688		1		1,749640288
	3		1,618644068		1		2,201848998
	4		1,988170347		1		4,163106796
	5		3,898492908		1		2,530916844
	6		2,481218698		2,352769679		3,273239437
	7		2,481218698		1,697452229		3,324858757
	8		2,012030075		1		3,405511811
	9		2,361904762		1		2,281967213
	10		3,125706215		1		2,679012346
	11		1,916342412		1		3,555944056
	12		2,107742728		1,936842105		2,795698925
	13		2,25607064		1,713068182		3,019543974
	14		1,92461308				1
	15		3,350515464				1
	16		3,056503198				0
Assay 2	1	5,444444444	4,676274945	1	1	1	1
	2	5,577220077	1,870754717	1	1	1	1
	3	7,688679245	1,699747687	1	2,17978459	1	1
	4	6,405825243	3,266996292	1	1,59057971	1	1,404718693
	5	4,178197065	1,652066116	1	1	1	2,588592233
	6	3,147482014	2,097729516	1	1	1	1,4
	7	2,083700441	1,971751412	1	1	1	1,735988201
	8	5,444444444	1	1	1	1	1,551446945
	9	5,577220077	1	1	1	1	2,638164755
	10	7,688679245	3,038095238	1	1		1
	11	6,405825243	2,976909414	1	1		1
	12	4,178197065	2,13864818	1	1		1,465686275
	13	8,28256513	3,35218254	1	1		1
	14	4,915492958	1,962859796	1	1		1
	15	4,665961945	2,408016444	1	1		1,306153846
	16		2,114563107		1		
	17		1,574683544		1		
	18				1		
	19				1		
	20				1		
	21				1		
	22				2,398181818		
	23				1		
	24				1		
Assay 3	1	5,557465585	3,516531889	1	1,264458852	1	1,981753249
	2	5,585363741	3,475675429	1,599281749	1,083694383	1,515479369	2,490979496
	3	4,626894576	3,67089239	2,363547398	1,058573419	1	1,621764484
	4	3,157243372	4,971968912	1	1	1	1,305900899
	5	7,508409262	4,150064778	1	1	1	1,578390148
	6	4,892316943	3,412449479	1	1	1	1,199817698
	7	5,925871949	1,871206634	3,818004699	1,827877939	1	1,274241837
	8	8,08871369	4,055509468	3,152251566	1,495331468	1	1,484340053
	9	10,11975388	3,563830193	2,853679092	1,493984038	1	1,702365888
	10	6,26253668	2,854662737	2,021858117	1	1	2,082054116
	11	6,086662962	3,239202796	4,708993065	1,316592075	1	1
	12	3,383170601	4,160203453	4,135688069	1	1	1,309030626
	13	7,372536864	3,673438459	1	1,236690411	1,570447229	1,32550812
	14	3,951608208		1	1,506119438	1,523347704	1,666696464
	15	3,634444917		1	1,949213882	1	1,99240686
	16	5,472491612		1,05934214	2,252530023	2,045147122	1,196458056
	17	4,944656384		1	1,271356137	3,174252676	1
	18	3,557375506		0,994506639	3,161525655	1,890570994	1,341625123
	19	4,368422239		1	1,847766537	1,935828613	1
	20	1		1,000535874	2,098531214	5,0362808	1,699701232
	21	5,743065562		1	1,530351045	1	1,405763271
	22	4,413582447			1,898222749	2,318725893	
	23	4,369174641			1,850058302	1,634016526	
	24	4,263158109			1,601112243	1,918580401	

Supplementary Figure 1

a)

The displayed blots in the MS were not cropped.

Antibody used:

Anti-HIS (rCPN0473)

Anti-GST (rLactC2 and rPLCgamma)

b)

Assay 1

	rLactC2		
DOPS GUV	+	-	total
1	6	0	6
2	14	0	14
3	8	0	8
4	2	0	2
5	12	0	12
6	9	1	10
7	8	0	8
total	59	1	60

	rLactC2		
DOPC GUV	+	-	total
1	0	19	19
2	0	28	28
3	0	30	30
total	0	77	77

Assay 2

	rLactC2		
DOPS GUVs	+	-	total
1	12	0	12
2	32	0	32
3	36	0	36
4	24	0	24
5	20	0	20
total	124	0	124

d)

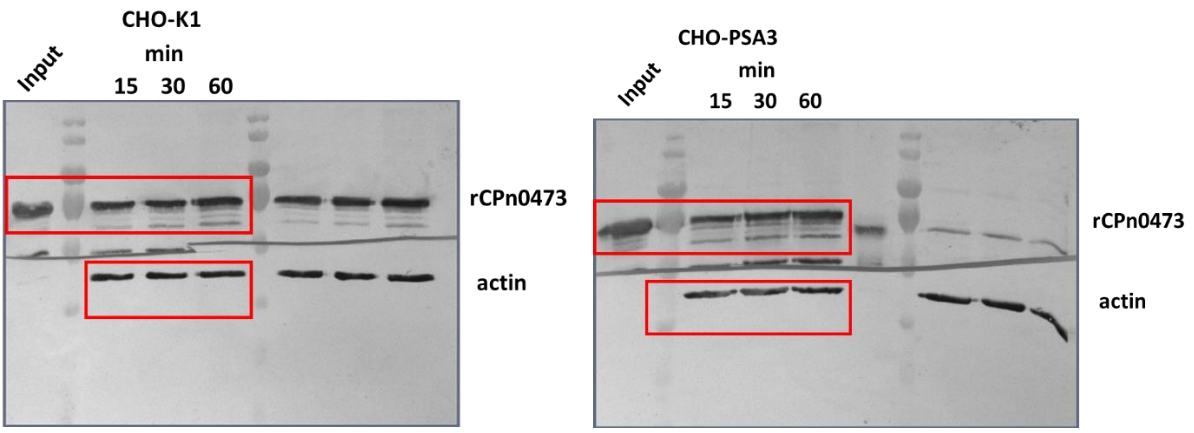

Antibodies used:

Anti-HIS (rCPn0473)

Anti-Actin (actin)

Supplementary Figure 4

e)

The table represents Alexa488 (LactC2)-intensities at single asymmetric GUVs, analyzed via ImageJ software. Background intensity is set to 1.

		DOPS inner leaflet, luminal Lact	DOPS inner leaflet, Lact C2 ou	DOPS outer leaflet, luminal LactC2, LIPP outside
Assay 1	1	2,29	1,00	1,00
	2	14,50	1,00	1,00
	3	2,69	1,00	1,00
	4	1,00	1,00	1,00
	5	1,00	1,00	1,00
	6	2,80	1,00	1,00
	7	3,90	1,00	1,00
	8	4,79	1,00	1,00
	9	4,44	1,00	1,00
	10	7,42	1,00	1,08
	11	7,37	1,00	1,00
	12	1,00	1,00	
	13		1,00	
	14		1,00	
	15		1,00	
Assay 2	1	9,02	1,00	1,00
	2	7,30	1,00	1,00
	3	9,70	1,00	1,69
	4	9,36	1,00	2,03
	5	12,47	1,00	1,00
	6	2,51	1,00	1,00
	7	3,58	1,00	2,12
	8	10,50	1,00	1,00
	9	9,11	1,00	1,68
	10	3,83	1,00	1,00
	11	7,70	1,00	1,45
	12	5,46	2,02	
	13	11,85	1,00	
	14	10,49	1,00	
	15		1,00	
	16		2,43	
	17		1,39	
	18		1,00	
Assay 3	1	5,971596416	1	1
	2	5,980575087	1,246505206	1
	3	5,176520923	1	1,241873913
	4	9,682314759	1	1
	5	5,088488849	1	1
	6	1,926948553	1	1
	7	1,411336621	1	1,391642596
	8	4,38403837	1	1
	9	8,986491094	1	1,315463491
	10	6,878994175	1	1
	11	6,319741036	1	
	12	3,623016816	1	
	13	2,40844946		
	14	1,617412109		
